# Maize Growth and Grain Yield Responses to a Micronized Humic Product Across Soil Types and Annual Weather Patterns in Central Iowa, United States

**DOI:** 10.3389/fpls.2021.672078

**Published:** 2021-05-12

**Authors:** Daniel C. Olk, Dana L. Dinnes, J. Rene Scoresby, Jerald W. Darlington, Charles R. Hurburgh, Glenn R. Rippke

**Affiliations:** ^1^National Laboratory for Agriculture and the Environment, United States Department of Agriculture – Agricultural Research Service, Ames, IA, United States; ^2^Minerals Technologies, Inc., New York, NY, United States; ^3^Currently Retired, Homedale, ID, United States; ^4^Department of Agricultural and Biosystems Engineering, Iowa State University, Ames, IA, United States

**Keywords:** humic product, grain yield, landscape, maize, soil type, variability

## Abstract

Despite growing interest in humic products as crop amendments, very few field evaluations have considered environmental factors of humic product efficacy. We determined the spatial and temporal variability in the efficacy of a micronized humic product on maize (*Zea mays* L.) growth and grain yield in two rainfed fields supporting a maize−soybean [*Glycine max* (L.) Merr.] rotation in 2012–2014, and 2016 in central Iowa, U.S. Crop management in both fields otherwise followed conventional farmer practices. In two dry growing seasons, mechanized combine measurements of grain yield increased significantly (*P* < 0.10) with humic product application on an eroded hilltop soil, amounting for two application rates to 930 and 1,600 kg ha^–1^ (11 and 19% of the control grain yield) in 2012, the droughtiest season, and 700 kg ha^–1^ (7% of the control) for the higher application rate in the somewhat droughty 2013 season. On a fertile side slope soil in the 2012 field, though, only a faint numeric response occurred in 2012, while on a toe slope soil the sole significant increase was in 2012, 870 kg ha^–1^ (14% increase above the control) for one application rate. With favorable rainfall in 2014 and 2016, significant grain yield increases with product application were small in the upland soil of 2014 and absent in 2016. Yield components analysis on 1-m row lengths of hand-collected samples attributed these yield boosts primarily to increased ear length, especially of the shorter ears. Combine grain yields, yield components, and total leaf area all demonstrated numerically slightly greater values for humic product treatments compared to the control in the vast majority of comparisons across years and soil types, with better distinction in the upland transects. Statistical significance, though, was reached only in the droughtier settings. The humic product had no consistent effects on nutrient concentrations of the grain, stover, or young leaves. Grain quality parameters showed a slight shift from protein to carbohydrates in the droughtier settings. Fifteen soil properties showed no response to the humic product. This humic product demonstrated the capability to improve maize growth in rainfed conditions in a high-yielding region, and its efficacy varied predictably with environmental conditions. This finding provides one potential explanation for inconsistent reports elsewhere of crop responses to humic products.

## Introduction

Humic products have received increasing attention as a potential field amendment for increasing crop growth and economic yield. Their efficacy in promoting plant growth has been demonstrated most commonly under controlled conditions (Chen and Aviad, 1990; Rose et al., 2014). A modest but increasing number of field studies has also demonstrated positive crop responses for horticultural (Canellas et al., 2015) and other agronomic and pasture crops (Verlinden et al., 2009; Calvo et al., 2014; Olk et al., 2018). These field studies have mostly involved only one or two site-year combinations. A smaller number of available studies reported no benefit of humic product application to crop growth in field settings (Hartz and Bottoms, 2010; Suddarth et al., 2019). The question then arises whether published studies represent only those intermittent cases where a positive response occurred, while an unknown number of unpublished trials failed to demonstrate any benefit. Information is lacking on the regularity of positive crop responses to humic products, especially under the range of environmental conditions that crops routinely encounter with field production.

Copious literature has demonstrated that agricultural amendments, including nitrogen (Cassman et al., 1996; Jaynes et al., 2004), other mineral fertilizers (Wollenhaupt et al., 1994; Havlin et al., 2013) and pesticides (Spark and Swift, 2002; Farenhorst, 2006) impact crop growth to varying degrees depending on local environmental and management factors. These can include crop type, soil type, compaction and other management-induced effects on soil properties, annual weather patterns, economic yield level, and tillage intensity. The efficacy of humic products might therefore also vary depending on these same factors, yet there have been no formal reports on such relationships.

In this study, we examined the field efficacy of a micronized humic product, Enersol^1^, created through extremely fine grinding of leonardite ore. Product efficacy was evaluated during four growing seasons in two production fields owned and managed by the same farm operator but in opposite phases of a maize [*Zea mays* (L.)−soybean *Glycine max* (L.) Merr.] annual crop rotation in central Iowa, United States. Both fields featured multiple soil types lying along elevational changes in spatial patterns that allowed experimental treatments to equally traverse all soil types. Annual precipitation varied among the 4 years from severe drought to highly favorable. We hypothesized that crop responses to the humic product would vary over space and time, as affected by soil type and annual weather patterns. In-season plant measurements were leaf area, by which the area of each leaf is presumed to reflect the favorability of growing conditions at the time that leaf developed (Eik and Hanway, 1965), and nutrient concentrations of young leaves, which are presumed to represent in-season availability of soil nutrients (Whitney et al., 1985). At crop physiological maturity, we measured yield components through hand-collected samples, followed by grain yield determination through mechanized combine.

## Materials and Methods

### Field Research Sites, Conditions and Operations

**FIGURE 1:**
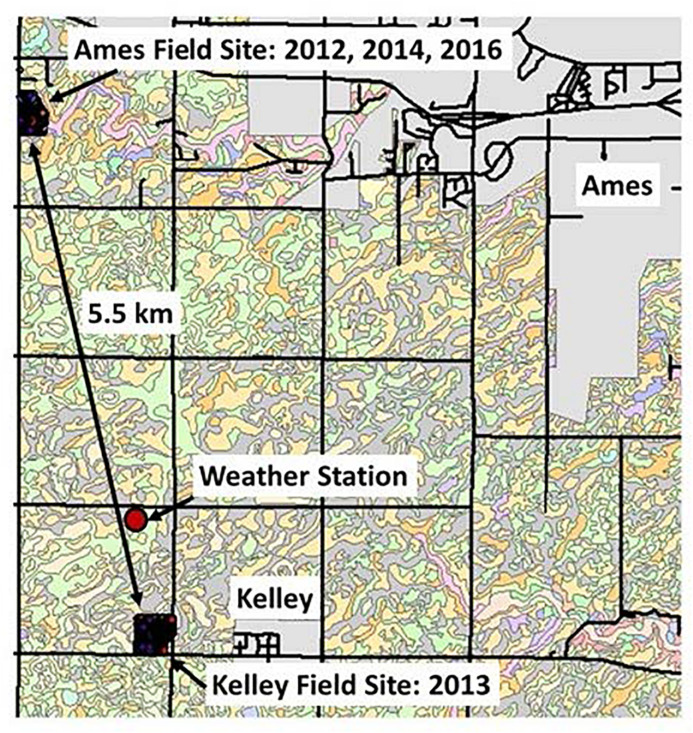
The Ames and Kelley field sites, shown with nearby roads, communities, soil type boundaries, and the USDA-ARS weather recording station.

Two on-farm sites for field research were located in central Iowa near Ames, Story County (42° 02′ N, 93° 37′ W)–one slightly west of Ames and another near Kelley, IA, United States, that were separated by a distance of 5.5 km (Figure 1). Both fields are located within the same watershed and thus have similar geology, soils, and climate, together with similar historic land use and farming practices, all of which were described by Eidem et al. (1999) and Hatfield et al. (1999).

Both fields were in a maize−soybean crop rotation in alternating years, and all analyses were conducted in the maize phase. Both sites are mapped within the Clarion (fine-loamy, mixed, superactive, mesic Typic Hapludoll)−Nicollet (fine-loamy, mixed, superactive, mesic Aquic Hapludoll)−Webster (fine-loamy, mixed, superactive, mesic Typic Endoaquoll) soil association, which is further described by Hatfield et al. (1999). Field-long treatment strips were specifically located at the primary site near Ames to include this continuum of the hilltop Clarion loam (2 to 5% slopes), sideslope Nicollet loam, and lowland Webster silty clay loam. At the site near Kelley, the field-long treatment strips included both the hilltop Clarion loam (5 to 9% slopes, moderately eroded) and a lowland pattern of the Canisteo silty clay loam (fine-loamy, mixed, superactive, calcareous, mesic Typic Endoaquoll) and Harps loam (fine-loamy, mixed, superactive, mesic Typic Calciaquoll). The soil mapping units for this Kelley field did not include sideslope soils. Clarion soils are characterized as being upland and well-drained. Nicollet soils occur on sideslopes, are somewhat poorly drained and typically are the most productive soils in the Clarion−Nicollet−Webster soil association, owing to favorable fertility and soil-water relations. Webster, Canisteo and Harps soils occur in flat areas and are all poorly drained. The predominant textural classes in their uppermost 100 cm are loam for the Clarion, loam to clay loam for the Nicollet, silty clay loam and clay loam for the Webster, silty clay loam, clay loam, and loam for the Canisteo, and loam, clay loam, and sandy clay loam for the Harps (Soil Conservation Service, 1985), indicating increasingly finer soil textures downslope. Sampling transects for hand collection of plant and soil samples were established in two areas designated as either upland or lowland landscape in both fields, omitting the sideslope Nicollet soil in the Ames field. Henceforth each landscape will be presumed as interchangeable with its respective soil type.

**FIGURE 2:**
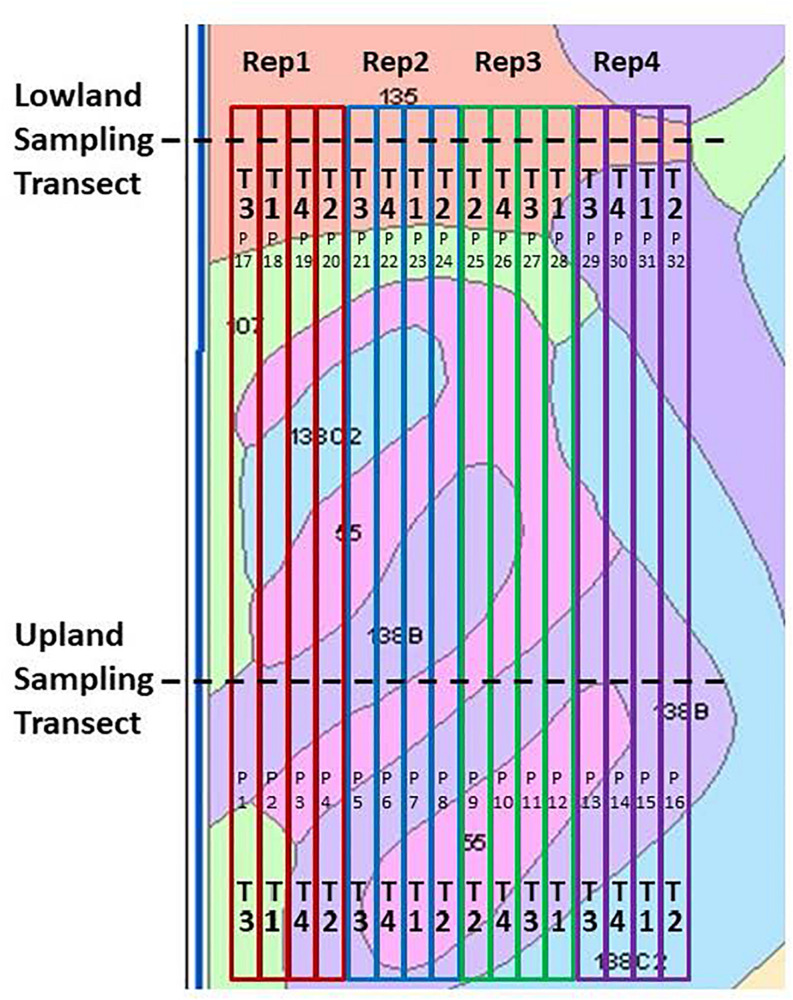
Field design of the Ames on-farm field research site location, shown with soil mapping units and boundaries of the field-long treatment strips. Key to treatments (“T”): T1, Control lacking Enersol humic product application; T2, lower rate of Enersol humic product application; T3, higher rate of humic product application and T4, a separate alkali-extracted humic product. Exact application rates changed among years, as explained in the text. Plot numbers are shown as “P.” Key to soil mapping units: 55, Nicollet loam, 1 to 3% slopes; 107, Webster silty clay loam, 0 to 2% slopes; 135, Coland clay loam, 0 to 2% slopes, 138B, Clarion loam, 2 to 5% slopes; and 138C2, Clarion loam, 5 to 9% slopes, moderately eroded.

**FIGURE 3:**
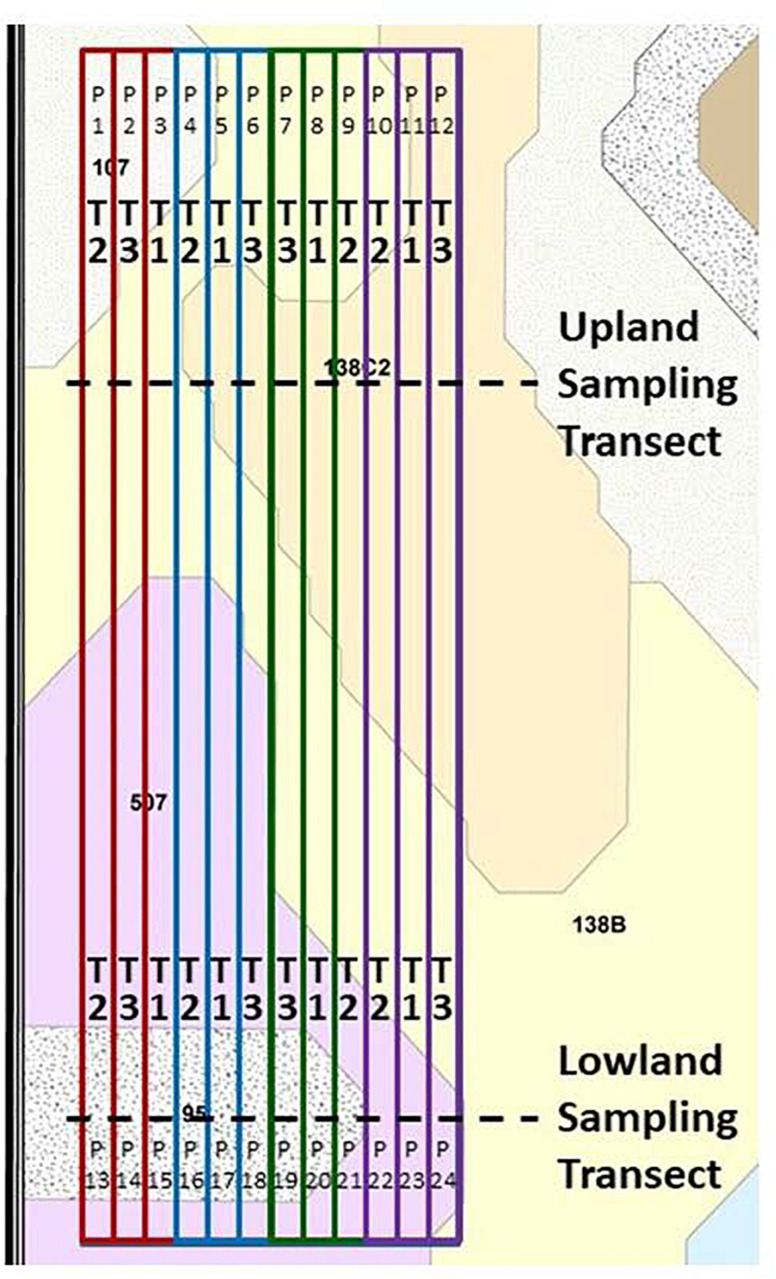
Field design of the Kelley on-farm field research site location, shown with soil mapping units and boundaries of the field-long treatment strips. The treatments are explained in the caption of Figure 2 and the main text. Plot numbers are shown as “P.” Key to soil mapping units: 95, Harps loam, 1 to 3% slopes; 138B, Clarion loam, 2 to 5% slopes; 138C2, Clarion loam, 5 to 9% slopes, moderately eroded; and 507, Canisteo silty clay loam, 0 to 2% slopes.

This study included the maize crop years of 2012, 2014, and 2016 for the field site near Ames (Figure 2) and the 2013 maize crop year for the field site near Kelley (Figure 3). The Kelley field was not used in 2015 due to its change to continuous maize beginning in 2014. Both experimental designs were imbedded within production fields operated by a commercial farming family, who followed their normal farming operations for the duration of this study. Planting dates, seed varieties and planting populations for the three maize years at the Ames site were as follows: 26 April 2012, Pioneer 453AM variety, 104-day relative maturity (RM) at 84,000 seeds ha^–1^; 23 April 2014, Pioneer 1151 AquaMax variety, 111-day RM at 85,000 seeds ha^–1^; and 26 April 2016, DeKalb 54-40RIB variety, 104-day RM at 84,000 seeds ha^–1^. Combine harvest dates for the Ames field were 01 October 2012, 13 November 2014, and 04 November 2016. At the site near Kelley, maize was planted on 18 May 2013, with DeKalb DKC62-97RIB variety and 112-day RM at 84,000 seeds ha^–1^. Combine harvest of the Kelley field was 14 November 2013. For each of the four maize seasons, the field received both chisel plow tillage after soybean harvest the previous autumn and a secondary chisel plow tillage operation in the spring prior to planting. Tillage operations were not conducted during the growing season. Chisel plow tillage was performed again in the autumn after maize harvest and in the following spring prior to soybean planting. Maize row spacing was always at 0.76 m, and all field-long treatment strip-plots were eight rows wide.

At the Ames field there were four replications of four humic product treatments, whose application rates and timings were recommended by the Enersol manufacturer. This humic product is created through media milling of a naturally occurring leonardite ore from Gascoyne, North Dakota (United States). The native pH of the ore is 3.5–5.0; thus, the milling generates an acidic, aqueous suspension concentrate. It contains about 28% leonardite solid particles, which include at least 180 g kg^–1^ humic acid, at least 15 g kg^–1^ fulvic acid, 4 g kg^–1^ S, and 4 g kg^–1^ Ca. Application timings are reported here following the leaf staging method that excludes the cotyledon leaf (Abendroth et al., 2011). The treatments in 2012 and 2014 were 2.5 L ha^–1^ Enersol humic product at the fourth maize leaf stage of vegetative growth (V4), 2 L ha^–1^ Enersol humic product at maize pre-emergence plus 1 L ha^–1^ Enersol humic product at V4, and 3 L ha^–1^ of a separate alkali-extracted humic product at V4, plus an unamended control (Figure 2). Based on crop responses to Enersol at other sites, in 2016 the application rates of the product were adjusted to 2.3 L ha^–1^ Enersol humic product at V4, 4.7 L ha^–1^ Enersol humic product at V4, and 4.7 L ha^–1^ of a separate alkali-extracted humic product at V4 (Figure 2). The alkali-extracted product treatment was more exploratory than were the other treatments, as the source of its extracted product varied among years. This treatment gave roughly analogous results as did the two Enersol treatments in the 3 years of the Ames field. Its presence in the field design affected the randomization of the other treatments within field replications, therefore it was included in statistical analyses of the whole field, including determination of main plot and soil type/landscape effects. Due to its variable sources, however, its results are not presented individually in this report.

At the Kelley field for 2013 (Figure 3), there were four replications of three treatments of the Ames field in 2012 and 2014, namely an untreated control, 2.5 L ha^–1^ humic product at V4, and 2 L ha^–1^ humic product at maize pre-emergence plus 1 L ha^–1^ humic product at V4.

The locations of the field-long strip plots and sampling transects in both fields were marked by global positioning system (GPS) and geographic information system (GIS) technologies in each year. Growing conditions were comparable among the field replicates within each transect except for the fourth field replicate in the upland transect of the Ames field, which was located on a less productive, eroded soil on a mild downward slope. In both fields, narrow walking paths were cut along the edges of the hand-sampling transects. All hand samples were collected at least 5 m distance from the cleared transect paths, within areas having uniform crop growth.

### Fertilization Rates and Timing

In the autumns prior to the spring plantings of the maize crops, N-phosphorus (P)-potassium (K) fertilizers were applied following soybean harvest at the respective rates of 112–90–134 kg ha^–1^. An additional 67 kg N ha^–1^ was added in the spring just prior to maize planting in conjunction with pre-plant herbicide application, totaling 179 kg N ha^–1^ applied to the fields for maize production in the years 2012 through 2014. This fertilization procedure changed in 2016, when approximately 4.9 Mg ha^–1^ of chicken (*Gallus gallus* domesticus) manure was applied to the Ames field on 4 April 2016, which was determined by later nutrient analyses to represent an effective N–P–K application rate of 28–67–56 kg ha^–1^. On 10 April 2016, an additional 134 kg N ha^–1^ of anhydrous ammonia (NH_4_^+^) was injected into the field, thus totaling 162 kg N ha^–1^ applied to the field for the 2016 maize growing season. No additional P and K was applied in 2016 beyond that contained in the chicken manure.

### Plant and Soil Sampling

The Ames field was not sampled for soil prior to the humic product application in 2012, due to fertilizer applications that preceded field layout. Initial soil samples were instead taken from each treatment strip on 11 September 2012 in conjunction with hand-sampling of maize yield components.

Maize grain yield was measured by mechanical combine equipped with yield-monitoring GPS and GIS technologies. The electronic yield data were analyzed to generate yield maps with overlays of the plots, sampling transects, soil types/landscapes, and areas of poor growth and crop damage that had been manually marked with GPS equipment during the growing season. All areas with damaged crop growth were excluded from further data processing and statistical analyses. Ten consecutive geo-referenced yield data points that were clearly located within each of the three soil mapping units (also representing the differing landscape positions) were identified and used to estimate the combine yield data, including adjustment to the standard equivalent of 15.5% market moisture.

Maize stover and ear samples were hand-harvested at physiological maturity each year for all treatment strips near both landscape sampling transects. In each treatment strip within each landscape transect (analogous to a plot), a one-row length of 1 m was harvested in areas of uniform growth by cutting seven evenly spaced plants at ground level. Four soil cores were taken to the 15 cm depth in the untrafficked interrows adjacent to the 1 m-hand-harvested row with a 3.18-cm diameter probe, composited within each plot, and stored at 4°C until later analyses for soil properties. Maize stover samples were oven-dried at 59°C under forced air, then immediately measured for oven-dry weights and mechanically shredded. Composite subsamples were taken of the shredded stover for later grinding through a Wiley mill (1 mm mesh screen) and then a Cyclone mill (Udy Corporation, Fort Collins, CO, United States) to a powder consistency. Maize ears were placed in plastic mesh bags and hung for drying before storage for subsequent measurements. All maize ear grains were later hand-shelled and passed through a mechanical seed counter for determination of 100-kernel weight. Total kernel weights of the hand samples were recorded and kernel moisture was recorded by a moisture meter. Maize grain moisture content was also determined by a standard oven-drying method (ASAE, 1988). Grain weight per 1-m row was then calculated and extrapolated to a hectare basis. For the 2012 to 2014 seasons, subsamples of harvested grains were initially air-dried to no more than 100 g kg^–1^ moisture content and then stored in airtight plastic bags until later analysis for protein, oil and starch contents using near-infrared spectroscopic (NIRS) procedures (Iowa Grain and Quality Initiative, 2004). Ear lengths from air-dried cobs were measured, and the cobs were then oven-dried for 3 days at 120°C and immediately measured for dry weight. The dried cob weights were then added to those of the 1-m stover samples to report total aboveground stover weight. Harvest index was defined as the ratio of grain weight to total aboveground stover weight.

In-season leaf samples are used to determine in-season plant nutrient status (Whitney et al., 1985) and were collected in 2012, 2013, and 2014 near the sampling transects at three key periods of nutrient uptake: V10-11, V14-15, and the maize kernel blister stage of reproductive growth, or “R2” (Abendroth et al., 2011). For the first two sampling times, the second uppermost leaf that had a visible collar was taken from 16 plants for each plot by soil type/landscape combination from areas of representative and uniform growth. The second leaf was chosen instead of the uppermost visible collar leaf to avoid instances in which the collar of the uppermost leaf had just become visible within the previous night such that N and other essential nutrients had not yet been transported to the leaf to its fullest potential. At the R2 sampling, the ear leaf was sampled.

Plant and soil samples were analyzed for pre-determined sets of properties as offered by a commercial laboratory. Plant tissue and grain total N analyses were performed through micro-Kjeldahl digestion and colorimetric determination of the extracted total N content. Plant tissue and grain analyses for all other nutrients (P, K, Mg, Ca, S, Zn, Mn, Cu, Fe, and B) were performed using wet digestion in nitric acid with 30% hydrogen peroxide and determination by inductively coupled plasma−mass spectrometry. Sodium and Al were also measured, but their results are not reported here due to their erratic and at times absent concentrations.

Methods for measuring soil extractable nutrients, pH, buffer pH, organic matter, and cation exchange capacity followed Denning et al. (1998). Soil pH was determined in a 1:1 (w:v) slurry in water, and buffer pH followed the Sikora Buffer method. Soil organic matter content was determined through loss on ignition. Available soil P was determined colorimetrically from a Bray 1 extraction (Bray and Kurtz, 1945). Extractants for other available soil nutrients included cadmium reduction (nitrate-N), 1 M ammonium acetate (K, Ca, Mg), monocalcium phosphate (S), diethylenetriamene pentaacetate (DTPA, Fe, Zn, Mn, Cu) and hot water (B).

In both transects and all four seasons, maize leaves were destructively measured for total leaf area on three consecutive plants that were evenly spaced and in areas of uniform growth near the sampling transects. Triplicate groups of three plants were marked at the V6 crop stage for three in-field samplings. The first leaf area measurement was at the V5 or V6 growth stage, when flagging tape was used to mark the internode between the V6 and V7 leaves of the other two plant sets. One of these sets was later used for the second measurement of leaf area at the V11 or V12 growth stage. On the remaining plant set, flagging tape was used to mark the internode between the V11 and V12 leaves for the final leaf area measurement soon after full tassel. For each leaf, its length and maximum width were measured to calculate leaf area by the method of Montgomery (1911) using the equation:

Leaf length (cm) × maximum leaf width (cm) × 0.75 = leaf area (cm^2^) (1)

Total plant leaf area was the sum of the areas from all leaves on each plant.

### Weather Recording Data Sites

Daily maximum and minimum temperatures (°C) and total rainfall (mm) were recorded at the 2-m height from a USDA-ARS weather station that was 4 km from the Ames site and 1.5 km from the Kelley site. These data were recorded for the period of 1 January 2012 through 31 December 2016. Each mean daily temperature was calculated as the mean of the daily maximum and daily minimum temperatures. Monthly mean high and low temperatures were calculated as the means of all daily values for their respective measures. The recent 30-year averages (1981–2010) for these same parameters were obtained from the U.S. Climate Data website for the Ames weather station, located at a distance of 6.5 km from the USDA weather station^2^.

### Statistical Analyses

Treatments were randomized by individual treatment strip within each replication, but not re-randomized by each soil type/landscape. Therefore, the experimental designs are treatments nested within treatment strips, and the program for SAS Proc Mixed (mixed models) program (SAS Institute, 2012) was accordingly adjusted to the proper degrees of freedom for this design. We used this program instead of the generalized linear models program for three reasons: (1) Proc Mix does not assume that observations are completely independent of each other, hence it tests for covariance and adjusts the levels of probability accordingly; (2) Proc Mix applies the same approach to errors; and (3) Proc Mix can allow the replication effect to be treated as a random variable instead of a fixed variable, which better represents field conditions. Repeated measures analyses, with time as an additional factor and with similarly adjusted degrees of freedom, were conducted for all data combined over multiple years from the Ames field, and for young leaf nutrient data, which were collected three times within each season at the same location. When time or soil type/landscape position were proven to be statistically significant factors, then additional analyses were conducted by individual time or soil type/landscape position to further examine treatment differences. Significance for all treatment and interaction terms was defined as *P* < 0.10. The Proc Corr procedure (SAS Institute, 2012) was used to correlate responses of several crop growth parameters to humic product application in the upland transect to an inverse index of drought stress. Lacking direct measurements of plant drought stress, we approximated drought stress as the ratio for each year of total rainfall from April to September in that year to the 30-year mean for total rainfall in those same months. We chose the Pearson correlation for this analysis. We did not attempt these correlations for the lowland transect, presuming a lack of correlation because this transect was less responsive to annual precipitation patterns than was the upland transect.

Several crop parameters showed consistent responses to the humic product that were not quite significant at *P* < 0.10 but would have been significant at less stringent thresholds. Due to their consistency, we also discuss these numeric trends, including the separation of results by soil type/landscape even in those cases having insignificant treatment−landscape interactions. We believe that to enable further refinement and development of site-specific, or precision, farming methods, all consistent information should be evaluated to improve production and environmental efficiencies of farming operations.

## Results

### Weather Patterns

Over the 4 years of this study, the weather patterns ranged from severe drought in 2012 to quite favorable in 2014, as indicated by deviations of monthly mean maximum and minimum temperatures and total monthly precipitation from the 30-year (1981–2010) averages (Table 1). The months of May through August comprise the bulk of the growing season for row crops in the temperate climate of central Iowa. In 2012, these 4 months coincided with the greatest period of precipitation deficits for all 4 years. Simultaneously, temperatures were notably higher than normal in March, May and July. Precipitation for this 4-month span was only 44% of normal, which, given the increased temperatures, caused readily apparent symptoms of severe moisture stress for the 2012 maize crop. On an annual basis, total 2012 precipitation (572 mm) was 37% less than the 30-year average (910 mm).

**TABLE 1:** Deviations from the 30-year average (1981–2010; Ames, IA, United States) for monthly maximum (*T*_*max*_) and minimum (*T*_*min*_) temperature and precipitation (Pre) for the 4 years of the study and its two field sites near Ames and Kelley, Story County, IA, United States.

	2012			2013			2014			2016		
Month	*T*_*max*_	*T*_*min*_	Pre	*T*_*max*_	*T*_*min*_	Pre	*T*_*max*_	*T*_*min*_	Pre	*T*_*max*_	*T*_*min*_	Pre
				
	^*o*^C		mm	^*o*^C		mm	^*o*^C		mm	^*o*^C		mm
January	4.0	3.3	−5	1.1	0.3	8	−2.1	–4.8	−13	–1.7	0.8	2
February	1.8	2.6	16	–0.8	0.4	10	−6.2	–7.5	23	0.4	2.9	3
March	8.8	6.8	−1	–6.3	–3.3	−5	−4.3	–3.3	−32	3.0	3.1	17
April	1.0	1.8	8	–4.2	–2.3	42	−2.2	–1.2	46	–0.7	0.8	−7
May	2.7	1.9	−55	–2.5	–0.2	110	−0.1	0.1	−47	–0.6	–0.3	−52
June	0.9	0.5	−60	–1.1	0.7	−45	−0.9	0.4	105	2.4	1.7	−97
July	3.7	1.4	−87	0.3	–0.5	−93	−3.2	–3.0	−56	–1.1	–0.3	0
August	1.0	−2.5	−72	1.3	0.2	−90	−1.2	0.7	52	–0.5	0.4	61
September	0.5	−3.2	−39	1.7	1.2	−42	−1.9	–0.9	0	1.3	2.5	99
October	−2.1	−2.0	−14	–1.4	–0.2	26	−0.7	–0.5	17	1.9	2.0	−49
November	2.4	0.4	−29	–1.8	–2.6	−15	−4.6	–4.7	−29	5.5	2.8	−17
December	1.9	1.2	2	–3.8	–3.8	−9	1.4	4.2	−3	–0.2	1.0	22
Annual	2.2	1.0	−338	–1.4	–0.8	−104	−2.2	–1.7	64	0.8	1.4	−17

In 2013, the spring was cooler than average, and May precipitation was 90% greater than average. With a planting date of 18 May, portions of the lowest-lying areas of the Kelley field were submerged early in the growing season, leading to their crop damage or loss. They were then excluded from sampling and calculation of grain yields. For the months of June through August, in contrast, precipitation was only 55% of normal, with near normal temperatures. Total annual precipitation in 2013 was 11% less than the 30-year average, and temperatures were slightly cooler than average.

In 2014 monthly precipitation varied within a narrow range and deviated little from normal means with just one exception; June experienced 83% greater precipitation than average. Therefore, growing conditions in 2014 were very favorable for crop production, except for extended conditions of overly wet soils in a portion of the lowland transect.

In 2016 a dry period in May and June provided 60% less precipitation than normal for that period. Yet for the entire year, precipitation was within 2% of normal and temperatures were close to normal. For subsequent interpretations of results, we consider 2012 as having severe drought, 2013 as wet early followed by moderate drought, 2014 as favorable throughout the growing season except for seasonal wetness in a portion of the lowland transect, and 2016 as moderate drought early followed by favorable throughout the remainder of the growing season.

### Combine Grain Yield

#### Ames Field (2012, 2014, 2016)

The only possible statistical analysis across multiple years was for 2012 and 2014, because the humic product rates and timing of application for the Ames site changed in 2016 from the earlier years, and a one-time application of chicken manure occurred shortly before the 2016 planting. In the combined analysis of 2012 and 2014, soil type/landscape, humic product treatment, and year effects and the soil type/landscape by year interaction were all highly significant (*P* < 0.0001). Non-significant interactions were found for treatment by soil type/landscape (*P* = 0.42), treatment by year (*P* = 0.75), and treatment by soil type/landscape by year (*P* = 0.99). Therefore, data will initially be presented by individual year and across the three soil types/landscapes.

Maize grain yields as measured by combine, and their statistical analyses for the Ames field site, are shown in Table 2, separately for 2012, 2014 and 2016. The field-averaged grain yield in 2012 was 9.4 Mg ha^–1^, 21% greater than the national average of 7.8 Mg ha^–1^ (USDA-National Agricultural Statistics Service, 2018). The severe drought in 2012 coincided with the most significant benefit of the humic product to maize grain yield. Across the three soil types/landscape positions in 2012, the 2.5 L ha^–1^ V4 treatment increased grain yield by 690 kg ha^–1^ (8%) compared to the control (8.86 Mg ha^–1^), and the 3 L ha^–1^ split application treatment increased yield by 780 kg ha^–1^ (9%). The main treatment effect was not significant (*P* = 0.24), but the soil type/landscape position effect was highly significant (*P* < 0.0001).

**TABLE 2:** Humic product maize grain yield responses to humic product application for the Ames on-farm field trial extracted as georeferenced data from combine yield maps and shown by year (2012, 2014 and 2016) and three soil type/landscape positions. Two rates of humic product application (H1 and H2) were compared to an unamended control (C).

2012						

	Maize grain yield (Mg ha^–1^)				Field-scale statistics	
		
Humic treatment	Upland soil^*a*^	Sideslope soil^*b*^	Lowland soil^*c*^	Mean		LSD Pr > F^*d*^
C	8.41	11.86	6.31	8.86	Humic treatment	0.24
H1^*e*^	9.34	12.12	7.18	9.55	Soil type/landscape	<0.0001
H2^*f*^	10.01	12.21	6.70	9.64	Treatment × soil type	0.86
**Mean**	9.25	12.06	6.73	9.35		

	**Humic treatment comparisons (LSD Pr > F)**					
		
	**Upland soil**	**Sideslope soil**	**Lowland soil**	**Field-scale**		

Main treatment	0.02	0.42	0.11	0.24		
C vs. H1	0.10	0.55	0.07	0.33		
C vs. H2	0.01	0.42	0.37	0.27		
H1 vs. H2	0.22	0.83	0.28	0.89		

**2014**						

	**Maize grain yield (Mg ha^–1^)**					
		
**Humic treatment**	**Upland soil**	**Sideslope soil**	**Lowland soil**	**Mean**		**LSD Pr > F**

C	12.08	12.77	12.78	12.54	Humic treatment	0.47
H1	12.65	12.42	12.97	12.68	Soil type/landscape	0.74
H2	13.10	12.60	12.92	12.87	Treatment × soil type	0.71
**Mean**	12.61	12.60	12.89	12.70		

	**Humic treatment comparisons (LSD Pr > F)**					
		
	**Upland soil**	**Sideslope soil**	**Lowland soil**	**Field-scale**		

Main treatment	0.07	0.45	0.49	0.47		
C vs. H1	0.24	0.39	0.50	0.71		
C vs. H2	0.05	0.66	0.61	0.38		
H1 vs. H2	0.35	0.67	0.87	0.61		

**2016**						

	**Maize grain yield (Mg ha^–1^)**					
		
**Humic treatment**	**Upland soil**	**Sideslope soil**	**Lowland soi**l	**Mean**		**LSD Pr > F**

C	13.92	14.07	14.58	14.19	Humic treatment	0.37
H1^*g*^	14.04	14.34	14.76	14.38	Soil type/landscape	0.02
H2^*h*^	13.98	14.77	14.82	14.52	Treatment × soil type	0.95
**Mean**	13.98	14.39	14.72	14.36		

	**Humic treatment comparisons (LSD Pr > F)**					
		
	**Upland soil**	**Sideslope soil**	**Lowland soil**	**Field-scale**		

Main treatment	0.64	0.42	0.58	0.37		
C vs. H1	0.58	0.70	0.67	0.57		
C vs. H2	0.79	0.31	0.59	0.32		
H1 vs. H2	0.77	0.52	0.90	0.66

*^a^Upland soil is the Clarion loam.*

*^b^Sideslope soil is the Nicollet loam.*

*^c^Lowland soil is the Webster silty clay loam.*

*^d^Probability of statistical significance as determined by the Least Significance Difference method.*

*^e^Enersol humic product broadcast applied in 2012 and 2014 at 2.5 L ha^–1^ at the fourth maize leaf stage.*

*^f^Enersol humic product broadcast split-applied in 2012 and 2014 at 2.0 L ha^–1^ at corn post-planting pre-emergence and 1 L ha^–1^ at the fourth maize leaf stage.*

*^g^Enersol humic product broadcast applied in 2016 at 2.3 L ha^–1^ at the fourth maize leaf stage.*

*^h^Enersol humic product broadcast applied in 2016 at 4.7 L ha^–1^ at the fourth maize leaf stage.*

Further statistical analysis by individual soil type/landscape showed significant main treatment effects on yield for the upland Clarion loam (*P* = 0.02). Yield increases above the control (8.41 Mg ha^–1^) there were 930 kg ha^–1^ (11%) for the 2.5 L ha^–1^ V4 treatment and 1.60 Mg ha^–1^ (19%) for the 3 L ha^–1^ split application treatment, and the corresponding levels of significance were 0.10 and 0.01, respectively. On the productive side slope Nicollet soil, there were slight numeric (2 and 3%) but insignificant increases by the humic product treatments above the control (overall soil mean 12.1 Mg ha^–1^). On the lowland Webster soil, the main treatment was nearly significant (*P* = 0.11). Comparing individual treatments to the control (6.31 Mg ha^–1^), the 2.5 L ha^–1^ V4 treatment had 870 kg ha^–1^ (14%) greater grain yield (*P* = 0.07), while the 3 L ha^–1^ split application treatment had only 390 kg ha^–1^ (6%) greater grain yield (*P* = 0.37). In all possible comparisons in 2012, grain yield did not differ significantly between the two humic treatments.

As opposed to the severe drought in 2012, growing conditions in 2014 were very favorable for crop production. Hence maize grain yield in 2014 was uniformly high across the Ames field, increasing above the corresponding 2012 yields by 33% for each humic treatment and by 42% for the control. Field-averaged grain yield was 12.7 Mg ha^–1^, 18% above the national average (USDA-National Agricultural Statistics Service, 2018). Effects were non-significant for the main treatment, soil type/landscape position and their interaction (Table 2). Across the three soil type/landscape positions, the 2.5 L ha^–1^ V4 treatment increased grain yield by only 1% compared to the control, and the 3 L ha^–1^ split application treatment increased yield by 3%, hence providing more muted responses than in 2012. Statistical analyses by individual soil type/landscape position found a significant (*P* = 0.07) treatment effect only for the upland Clarion soil: the 3 L ha^–1^ split application treatment had significantly greater (*P* = 0.05) grain yield than did the control (12.1 Mg ha^–1^) by 8%. The 2.5 L ha^–1^ V4 treatment had 5% greater grain yield than did the control for the upland Clarion soil, but the increase was not significant (*P* = 0.24). For the side slope Nicollet soil, both humic treatments had slight yield decreases compared to the control, although neither was significant (overall soil mean 12.6 Mg ha^–1^). For the lowland Webster soil, slight yield gains with both humic product treatments were non-significant compared to the control (overall soil mean 12.9 Mg ha^–1^).

Following manure application in early 2016, maize grain yields in 2016 increased by 13% above the 2014 grain yields for each humic product treatment and the control. Humic treatment effects in 2016 remained muted. Field-averaged grain yield was 14.4 Mg ha^–1^, 31% above the national average (USDA-National Agricultural Statistics Service, 2018). Main treatment and its interaction with soil type/landscape were non-significant, but soil type/landscape had a significant (*P* = 0.02) effect, due to lower grain yields in the upland Clarion soil. Across the three soil type/landscape positions, the 2.3 L ha^–1^ treatment increased grain yield by only 1% above the control and the 4.7 L ha^–1^ treatment increased yield by 2%. Statistical analyses by individual soil type/landscape position found no significant treatment effects, although the grain yield increased numerically above the control for each humic treatment in each soil type/landscape position.

#### Kelley Field (2013)

In the single year of humic product treatments in the Kelley field (2013), the field-averaged grain yield was 11.1 Mg ha^–1^, 10% above the national average (USDA-National Agricultural Statistics Service, 2018) and 19% greater than for the 2012 Ames field but 13% less than for the 2014 Ames field. Similar to 2016, the main treatment and its interaction with soil type/landscape position had non-significant effects on grain yield while soil type/landscape again had a significant (*P* < 0.0001) effect due to lower grain yields in the upland Clarion soil (Table 3). Field observations attribute this decrease to the droughty conditions that prevailed after early growth stages. Across both soil type/landscape positions, the 2.5 L ha^–1^ V4 treatment decreased grain yield by 3% compared to the control while the 3 L ha^–1^ split application treatment increased yield by 3%. This paradox was resolved through statistical analyses by individual soil type/landscape position, which found numerically positive grain yield responses to both humic treatments in the upland Clarion loam, including a significant (*P* = 0.08) increase by 700 kg ha^–1^ (7%) with the 3 L ha^–1^ split application treatment compared to the control (9.5 Mg ha^–1^). But in the lowland Canisteo silty clay loam/Harps loam complex, which encountered early season flooding, grain yield either decreased by 6% (*P* = 0.18) with the 2.5 L ha^–1^ V4 treatment compared to the control (12.6 Mg ha^–1^), or it was unresponsive to the 3 L ha^–1^ split application treatment.

**TABLE 3:** Maize grain yield responses to humic product application for the 2013 Kelley on-farm field trial, extracted as georeferenced data from a combine yield map and shown by three soil type/landscape positions.

	Maize grain yield (Mg ha^–1^)		
	
Treatment	Upland, Clarion soil	Lowland Canisteo/Harps soils	Mean
Control	9.53	12.56	11.04
Humic 1^a^	9.69	11.81	10.75
Humic 2^b^	10.23	12.56	11.40
**Mean**	9.82	12.31	11.06

**Statistical analyses**			

	**Whole field**		
	
	**LSD Pr > F**	**Comparisons**	**LSD Pr > F**^*c*^

Treatment	0.39	Control vs. Humic 1	0.53
Soil type/landscape	<0.0001	Control vs. Humic 2	0.45
Treatment × soil type	0.59	Humic 1 vs. Humic 2	0.18

	**Upland Clarion soil**		
	
	**LSD Pr > F**	**Comparisons**	**LSD Pr > F**

Treatment	0.17	Control vs. Humic 1	0.65
		Control vs. Humic 2	0.08
		Humic 1 vs. Humic 2	0.16

	**Lowland Canisteo/Harps soils**		
	
	**LSD Pr > F**	**Comparisons**	**LSD Pr > F**

Treatment	0.28	Control vs. Humic 1	0.18
		Control vs. Humic 2	0.99
		Humic 1 vs. Humic 2	0.17

*Two rates of humic product application (Humic 1 and Humic 2) were compared to an unamended control (C).*

*^a^Enersol humic product broadcast applied at 2.5 L ha^–1^ at the fourth maize leaf stage.*

*^b^Enersol humic product broadcast split-applied at 2.0 L ha^–1^ post-planting before maize emergence and 1 L ha^–1^ at the fourth maize leaf stage.*

*^c^Probability of statistical significance as determined by the Least Significance Difference method.*

Summarizing across both fields and the 4 years, combine-measured grain yield numerically increased with application of a humic product compared to the control in 18 of 22 comparisons for either humic product treatment in a specific soil type/landscape position and in a single year. These increases were commonly modest, and their statistical significances were affected by year or soil type/landscape position.

### Yield Components

#### Ames Field (2012, 2014, 2016)

Field-scale grain weights were calculated from the 1-m yield component samples for the upland Clarion and lowland Webster soils. These estimates consistently exceeded the combine-generated grain yields, as yield component samples were collected in areas of healthy crop growth, avoiding missing or damaged plants.

In the droughty 2012 season, cob length responded significantly to both main treatment (*P* = 0.08) and soil type/landscape (*P* = 0.07) for the whole field, and 100-kernel weight responded significantly to soil type/landscape (*P* = 0.02), while main treatment, soil type/landscape, and their interaction did not significantly affect whole field grain weights, stover weights or harvest index (Table 4). Statistical analyses by individual soil type/landscape position found the only significant responses to humic product application were positive for both grain weight (14.0 Mg ha^–1^ vs. 12.1 Mg ha^–1^ for the control) and cob length (17.1 cm vs. 16.0 cm for the control) for the 3 L ha^–1^ split application treatment in the upland soil. Summarizing all five yield components and both humic product application rates in 2012, the levels of significance for crop response to either humic product application were numerically stronger (smaller *P*-values) in the upland soil than in the lowland soil in seven of 10 cases.

**TABLE 4:** Maize yield component responses to humic product application for the Ames on-farm field trial (2012, 2014, and 2016).

2012							

Grain weight (Mg ha^–^^1^)							

Treatment	Upland^*a*^	Lowland^*b*^	Mean	Statistics	LSD Pr > F^*c*^	Tests	LSD Pr > F
C	12.11	13.00	12.56	Treatment	0.48	C vs. H1	0.80
H1^*d*^	12.39	13.47	12.93	Landscape	0.40	C vs. H2	0.34
H2^*e*^	13.99	13.26	13.62	Interaction	0.67	H1 vs. H2	0.47
Mean	12.83	13.24					

	**By upland**				**By lowland**		
	**Tests**	**LSD Pr > F**			**Tests**	**LSD Pr > F**	

	C vs. H1	0.89			C vs. H1	0.68	
	C vs. H2	0.04			C vs. H2	0.85	
	H1 vs. H2	0.05			H1 vs. H2	0.82	

**Cob length (cm)**							

**Treatment**	**Upland**	**Lowland**	**Mean**	**Statistics**	**LSD Pr > F**	**Tests**	**LSD Pr > F**

C	16.01	16.74	16.38	Treatment	0.08	C vs. H1	0.15
H1	16.78	17.46	17.12	Landscape	0.07	C vs. H2	0.12
H2	17.11	17.23	17.17	Interaction	0.81	H1 vs. H2	0.92
Mean	16.63	17.14					

	**By upland**				**By lowland**		
	**Tests**	**LSD Pr > F**			**Tests**	**LSD Pr > F**	

	C vs. H1	0.15			C vs. H1	0.26	
	C vs. H2	0.05			C vs. H2	0.44	
	H1 vs. H2	0.52			H1 vs. H2	0.71	

**Stover weight (Mg ha**^–^**^1^)**							

**Treatment**	**Upland**	**Lowland**	**Mean**	**Statistics**	**LSD Pr > F**	**Tests**	**LSD Pr > F**

C	9.57	10.51	10.04	Treatment	0.38	C vs. H1	0.57
H1	10.18	10.78	10.48	Landscape	0.20	C vs. H2	0.35
H2	10.64	10.75	10.70	Interaction	0.87	H1 vs. H2	0.72
Mean	10.13	10.68					

	**By upland**				**By lowland**		
	**Tests**	**LSD Pr > F**			**Tests**	**LSD Pr > F**	

	C vs. H1	0.62			C vs. H1	0.44	
	C vs. H2	0.31			C vs. H2	0.47	
	H1 vs. H2	0.58			H1 vs. H2	0.96	

**Harvest index**							

**Treatment**	**Upland**	**Lowland**	**Mean**	**Statistics**	**LSD Pr > F**	**Tests**	**LSD Pr > F**

C	1.07	1.05	1.06	Treatment	0.89	C vs. H1	0.81
H1	1.04	1.07	1.05	Landscape	0.79	C vs. H2	0.64
H2	1.12	1.05	1.09	Interaction	0.77	H1 vs. H2	0.47
Mean	1.08	1.06					

	**By upland**				**By lowland**		
	**Tests**	**LSD Pr > F**			**Tests**	**LSD Pr > F**	

	C vs. H1	0.38			C vs. H1	0.85	
	C vs. H2	0.28			C vs. H2	0.98	
	H1 vs. H2	0.07			H1 vs. H2	0.87	

**100-Kernel Wt (g)**							

**Treatment**	**Upland**	**Lowland**	**Mean**	**Statistics**	**LSD Pr > F**	**Tests**	**LSD Pr > F**

C	27.78	26.20	26.99	Treatment	0.42	C vs. H1	0.56
H1	26.86	26.24	26.55	Landscape	0.02	C vs. H2	0.42
H2	27.52	25.22	26.37	Interaction	0.63	H1 vs. H2	0.81
Mean	27.39	25.89					

	**By upland**				**By lowland**		
	**Tests**	**LSD Pr > F**			**Tests**	**LSD Pr > F**	

	C vs. H1	0.36			C vs. H1	0.95	
	C vs. H2	0.79			C vs. H2	0.17	
	H1 vs. H2	0.51			H1 vs. H2	0.16	

**2014**							

**Grain weight (Mg ha**^–^**^1^)**							

**Treatment**	**Upland**	**Lowland**	**Mean**	**Statistics**	**LSD Pr > F**	**Tests**	**LSD Pr > F**

C	16.20	17.24	16.72	Treatment	0.35	C vs. H1	0.34
H1	16.72	17.98	17.35	Landscape	0.03	C vs. H2	0.49
H2	16.79	17.57	17.18	Interaction	0.86	H1 vs. H2	0.79
Mean	16.57	17.60					

	**By upland**				**By lowland**		
	**Tests**	**LSD Pr > F**			**Tests**	**LSD Pr > F**	

	C vs. H1	0.48			C vs. H1	0.42	
	C vs. H2	0.43			C vs. H2	0.72	
	H1 vs. H2	0.93			H1 vs. H2	0.65	

**Cob length (cm)**							

**Treatment**	**Upland**	**Lowland**	**Mean**	**Statistics**	**LSD Pr > F**	**Tests**	**LSD Pr > F**

C	16.01	16.43	16.22	Treatment	0.29	C vs. H1	0.26
H1	16.46	16.48	16.47	Landscape	0.64	C vs. H2	0.46
H2	16.40	16.36	16.38	Interaction	0.57	H1 vs. H2	0.68
Mean	16.29	16.43					

	**By upland**				**By lowland**		
	**Tests**	**LSD Pr > F**			**Tests**	**LSD Pr > F**	

	C vs. H1	0.12			C vs. H1	0.89	
	C vs. H2	0.18			C vs. H2	0.84	
	H1 vs. H2	0.80			H1 vs. H2	0.74	

**Stover weight (Mg ha**^–^**^1^)**							

**Treatment**	**Upland**	**Lowland**	**Mean**	**Statistics**	**LSD Pr > F**	**Tests**	**LSD Pr > F**

C	11.33	11.91	11.62	Treatment	0.55	C vs. H1	0.42
H1	11.67	12.45	12.06	Landscape	0.03	C vs. H2	0.83
H2	11.19	12.28	11.74	Interaction	0.94	H1 vs. H2	0.55
Mean	11.40	12.21					

	**By upland**				**By lowland**		
	**Tests**	**LSD Pr > F**			**Tests**	**LSD Pr > F**	

	C vs. H1	0.56			C vs. H1	0.50	
	C vs. H2	0.81			C vs. H2	0.64	
	H1 vs. H2	0.41			H1 vs. H2	0.83	

**Harvest index**							

**Treatment**	**Upland**	**Lowland**	**Mean**	**Statistics**	**LSD Pr > F**	**Tests**	**LSD Pr > F**

C	1.22	1.24	1.23	Treatment	0.74	C vs. H1	0.96
H1	1.22	1.23	1.22	Landscape	0.58	C vs. H2	0.53
H2	1.28	1.22	1.25	Interaction	0.62	H1 vs. H2	0.49
Mean	1.24	1.23					

	**By upland**				**By lowland**		
	**Tests**	**LSD Pr > F**			**Tests**	**LSD Pr > F**	

	C vs. H1	0.91			C vs. H1	0.80	
	C vs. H2	0.12			C vs. H2	0.69	
	H1 vs. H2	0.14			H1 vs. H2	0.88	

**100-Kernel Wt (g)**							

**Treatment**	**Upland**	**Lowland**	**Mean**	**Statistics**	**LSD Pr > F**	**Tests**	**LSD Pr > F**

C	24.56	25.17	24.86	Treatment	0.38	C vs. H1	0.33
H1	24.87	26.92	25.89	Landscape	0.08	C vs. H2	0.58
H2	25.21	25.68	25.44	Interaction	0.76	H1 vs. H2	0.67
Mean	24.88	25.92					

	**By upland**				**By lowland**		
	**Tests**	**LSD Pr > F**			**Tests**	**LSD Pr > F**	

	C vs. H1	0.78			C vs. H1	0.22	
	C vs. H2	0.56			C vs. H2	0.71	
	H1 vs. H2	0.76			H1 vs. H2	0.38	

**2016**							

**Grain weight (Mg ha**^–^**^1^)**							

Treatment	Upland	Lowland	Mean	**Statistics**	**LSD Pr > F**	**Tests**	**LSD Pr > F**

C	16.38	17.45	16.92	Treatment	0.58	C vs. H1	0.61
H1^*f*^	17.01	17.39	17.20	Landscape	0.02	C vs. H2	0.66
H2^*g*^	17.39	16.95	17.17	Interaction	0.08	H1 vs. H2	0.95
Mean	16.93	17.26					

	**By upland**				**By lowland**		
	**Tests**	**LSD Pr > F**			**Tests**	**LSD Pr > F**	

	C vs. H1	0.34			C vs. H1	0.89	
	C vs. H2	0.15			C vs. H2	0.41	
	H1 vs. H2	0.59			H1 vs. H2	0.49	

**Cob length (cm)**							

**Treatment**	**Upland**	**Lowland**	**Mean**	**Statistics**	**LSD Pr > F**	**Tests**	**LSD Pr > F**
C	17.4	18.3	17.8	Treatment	0.21	C vs. H1	0.21
H1	18.0	18.8	18.4	Landscape	<0.01	C vs. H2	0.36
H2	18.2	18.2	18.2	Interaction	0.19	H1 vs. H2	0.70
Mean	17.9	18.4					

	**By upland**				**By lowland**		
	**Tests**	**LSD Pr > F**			**Tests**	**LSD Pr > F**	

	C vs. H1	0.23			C vs. H1	0.34	
	C vs. H2	0.11			C vs. H2	0.88	
	H1 vs. H2	0.63			H1 vs. H2	0.27	

**Stover weight (Mg ha**^–^**^1^)**							

**Treatment**	**Upland**	**Lowland**	**Mean**	**Statistics**	**LSD Pr > F**	**Tests**	**LSD Pr > F**

C	9.26	10.51	9.88	Treatment	0.64	C vs. H1	0.50
H1	9.98	10.29	10.13	Landscape	0.05	C vs. H2	0.88
H2	10.20	9.68	9.93	Interaction	0.10	H1 vs. H2	0.60
Mean	9.81	10.16					

	**By upland**				**By lowland**		
	**Tests**	**LSD Pr > F**			**Tests**	**LSD Pr > F**	

	C vs. H1	0.12			C vs. H1	0.71	
	C vs. H2	0.05			C vs. H2	0.17	
	H1 vs. H2	0.60			H1 vs. H2	0.29	

**Harvest Index**							

**Treatment**	**Upland**	**Lowland**	**Mean**	**Statistics**	**LSD Pr > F**	**Tests**	**LSD Pr > F**

C	1.51	1.42	1.47	Treatment	0.96	C vs. H1	0.63
H1	1.46	1.45	1.45	Landscape	0.23	C vs. H2	0.70
H2	1.46	1.50	1.48	Interaction	0.19	H1 vs. H2	0.39
Mean	1.48	1.46					

	**By upland**				**By lowland**		
	**Tests**	**LSD Pr > F**			**Tests**	**LSD Pr > F**	

	C vs. H1	0.12			C vs. H1	0.62	
	C vs. H2	0.10			C vs. H2	0.10	
	H1 vs. H2	0.94			H1 vs. H2	0.21	

**100-Kernel Wt (g)**							

**Treatment**	**Upland**	**Lowland**	**Mean**	**Statistics**	**LSD Pr > F**	**Tests**	**LSD Pr > F**

C	28.70	29.48	29.10	Treatment	0.73	C vs. H1	0.75
H1	29.17	29.40	29.28	Landscape	0.44	C vs. H2	0.77
H2	29.02	29.53	29.27	Interaction	0.86	H1 vs. H2	0.98
Mean	28.96	29.47					

	**By upland**				**By lowland**	
	**Tests**	**LSD Pr > F**			**Tests**	**LSD Pr > F**

	C vs. H1	0.61			C vs. H1	0.89
	C vs. H2	0.73			C vs. H2	0.95
	H1 vs. H2	0.87			H1 vs. H2	0.85

*Two rates of humic product application (H1 and H2) were compared to an unamended control (C) for two landscapes/soil types.*

*^a^Clarion loam.*

*^b^Webster silty clay loam.*

*^c^Probability of statistical significance as determined by the Least Significance Difference method.*

*^d^Enersol humic product broadcast applied in 2012 and 2014 at 2.5 L ha^–1^ at the maize fourth leaf stage.*

*^e^Enersol humic product broadcast split-applied in 2012 and 2014 at 2.0 L ha^–1^ at maize post-planting before emergence and 1 L ha^–1^ at the maize fourth leaf stage.*

*^f^Enersol humic product broadcast applied in 2016 at 2.3 L ha^–1^ at the maize fourth leaf stage.*

*^g^Enersol humic product broadcast applied in 2016 at 4.7 L ha^–1^ at the maize fourth leaf stage.*

In the favorable 2014 season, main treatment had no significant effects on any of the five yield component parameters for the whole field, but soil type/landscape significantly affected grain weight, stover weight and 100-kernel weight because of smaller values in the upland soil. Statistical analyses by individual soil type/landscape position found no significant responses to the humic product for any of the yield components, although for the upland landscape positive responses by cob length to the 2.5 L ha^–1^ V4 treatment and harvest index to the 3 L ha^–1^ split application treatment (both *P* = 0.12) neared the 0.10 threshold. Across all five yield components, the upland soil had numerically stronger levels of significance with humic product application than did the lowland soil in only five of 10 cases.

In the 2016 season, the humic main treatment did not significantly affect any of the five yield components for the whole field, while soil type/landscape significantly affected grain weight, cob length, and stover weight, due to mostly lower values in the upland soil. The soil type/landscape by main treatment interaction significantly affected grain weight and nearly significantly affected stover weight (*P* = 0.10). Statistical analyses by individual soil type/landscape position found the only significant increase with humic product application was for stover weight for the 4.7 L ha^–1^ treatment in the upland transect (10.2 Mg ha^–1^ vs. 9.3 Mg ha^–1^). Yet this application rate also approached the *P* = 0.10 threshold of significance for grain weight, cob length, and harvest index in the upland soil and harvest index in the lowland soil, while the 2.3 L ha^–1^ treatment approached significance in the upland landscape for stover weight and harvest index. Across all five yield components, levels of significance with humic product application were numerically stronger (smaller *P*-values) in the upland soil than in the lowland soil in nine of 10 cases, with identical values in the 10th case. Most numeric differences were large.

#### Kelley Field (2013)

In the single year of humic product treatments in the Kelley field (2013), the main treatment and soil type/landscape both significantly affected grain weight, cob length, and harvest index, while soil type/landscape also significantly affected stover weight and 100-kernel weight (Table 5). These trends reflect yet more positive crop responses to humic product application in the lowland transect than in the upland transect. The main treatment by soil type/landscape interaction was significant only for stover weight. Statistical analyses by soil type/landscape position found significant increases for both humic application rates in the lowland transect for grain weight, cob length, and stover weight and also for 100-kernel weight for the 2.5 L ha^–1^ application treatment. In the upland transect, the sole response nearing significance was by harvest index to the 3 L ha^–1^ split application treatment (*P* = 0.10). Across all five yield components, the levels of significance were numerically stronger in the upland soil than in the lowland soil in only two of 10 cases. These trends are inconsistent with the combine grain yields, where the upland soil responded positively to the humic product and the lowland soil responded generally negatively. The lowland yield component samples were collected at slightly higher elevations than were the lowland combine yield data, probably lessening the growth limitations caused by early season wet conditions. Also, the elevational difference between upland and lowland was smaller in this field than in the Ames field.

**TABLE 5:** Maize yield component responses to humic product application for the Kelley on-farm field trial (2013).

Grain weight (Mg ha^–^^1^)							

Treatment	Upland^*a*^	Lowland^*b*^	Mean	Statistics:	LSD Pr > F^*c*^	Tests	LSD Pr > F
C	15.00	15.04	15.02	Treatment	0.04	C vs. H1	0.02
H1^*d*^	16.20	19.00	17.60	Landscape	0.04	C vs. H2	0.03
H2^*e*^	16.13	18.58	17.35	Interaction	0.28	H1 vs. H2	0.79
Mean	15.78	17.54	16.66				

	**By upland**				**By lowland**		
	**Tests**	**LSD Pr > F**			**Tests**	**LSD Pr > F**	

	C vs. H1	0.25			C vs. H1	0.01	
	C vs. H2	0.28			C vs. H2	0.02	
	H1 vs. H2	0.94			H1 vs. H2	0.73	

**Cob length (cm)**							

**Treatment**	**Upland**	**Lowland**	**Mean**	**Statistics:**	**LSD Pr > F**	**Tests**	**LSD Pr > F**

C	16.98	17.19	17.08	Treatment	0.08	C vs. H1	0.04
H1	17.60	19.27	18.43	Landscape	0.07	C vs. H2	0.01
H2	17.47	18.94	18.20	Interaction	0.81	H1 vs. H2	0.27
Mean	17.35	18.46	17.90				

	**By upland**				**By lowland**		
	**Tests**	**LSD Pr > F**			**Tests**	**LSD Pr > F**	

	C vs. H1	0.35			C vs. H1	<0.01	
	C vs. H2	0.45			C vs. H2	<0.01	
	H1 vs. H2	0.85			H1 vs. H2	0.48	

**Stover weight (Mg ha**^–^**^1^)**							

**Treatment**	**Upland**	**Lowland**	**Mean**	**Statistics:**	**LSD Pr > F**	**Tests**	**LSD Pr > F**

C	10.10	10.60	10.35	Treatment	0.17	C vs. H1	0.09
H1	10.44	12.76	11.60	Landscape	0.07	C vs. H2	0.13
H2	10.17	12.74	11.46	Interaction	0.05	H1 vs. H2	0.72
Mean	10.24	12.04	11.14				

	**By upland**				**By lowland**		
	**Tests**	**LSD Pr > F**			**Tests**	**LSD Pr > F**	

	C vs. H1	0.66			C vs. H1	0.02	
	C vs. H2	0.92			C vs. H2	0.02	
	H1 vs. H2	0.58			H1 vs. H2	0.96	

**Harvest index**							

**Treatment**	**Upland**	**Lowland**	**Mean**	**Statistics:**	**LSD Pr > F**	**Tests**	**LSD Pr > F**

C	1.26	1.20	1.23	Treatment	0.09	C vs. H1	0.06
H1	1.31	1.26	1.28	Landscape	<0.01	C vs. H2	0.05
H2	1.34	1.24	1.29	Interaction	0.69	H1 vs. H2	0.97
Mean	1.30	1.23	1.27				

	**By upland**				**By lowland**		
	**Tests**	**LSD Pr > F**			**Tests**	**LSD Pr > F**	

	C vs. H1	0.23			C vs. H1	0.13	
	C vs. H2	0.10			C vs. H2	0.29	
	H1 vs. H2	0.57			H1 vs. H2	0.55	

**100-Kernel Wt (g)**							

**Treatment**	**Upland**	**Lowland**	**Mean**	**Statistics:**	**LSD Pr > F**	**Tests**	**LSD Pr > F**

C	27.35	28.74	28.04	Treatment	0.22	C vs. H1	0.13
H1	28.41	31.98	30.20	Landscape	0.04	C vs. H2	0.14
H2	29.06	31.13	30.10	Interaction	0.67	H1 vs. H2	0.94
Mean	28.27	30.62	29.44				

	**By upland**				**By lowland**		
	**Tests**	**LSD Pr > F**			**Tests**	**LSD Pr > F**	

	C vs. H1	0.39			C vs. H1	0.09	
	C vs. H2	0.18			C vs. H2	0.19	
	H1 vs. H2	0.59			H1 vs. H2	0.62	

*Two rates of humic product application (H1 and H2) were compared to an unamended control (C) for two landscape positions.*

*^a^Clarion loam.*

*^b^Canisteo silty clay loam/Harps loam.*

*^c^Probability of statistical significance as determined by the Least Significance Difference method.*

*^d^Enersol humic product broadcast applied at 2.5 L ha^–1^ at the fourth maize leaf stage.*

*^e^Enersol humic product broadcast split-applied at 2.0 L ha^–1^ post-planting before maize emergence and 1 L ha^–1^ at the fourth maize leaf stage.*

Although yield component responses to humic product application were frequently non-significant, the responses were mostly numerically positive. For each yield component, of the 16 comparisons between humic product application and the control for all 4 years, both humic product rates, and both transects, numerically positive responses to the product occurred for grain weight and cob length in 14 cases each, for stover weight in 13 cases, for 100-kernel weight in 12 cases, but for harvest index only in nine cases. Similar to the combine-based grain yields, these increases were mostly modest, and their statistical significances were affected by year and soil type/landscape position.

**FIGURE 4:**
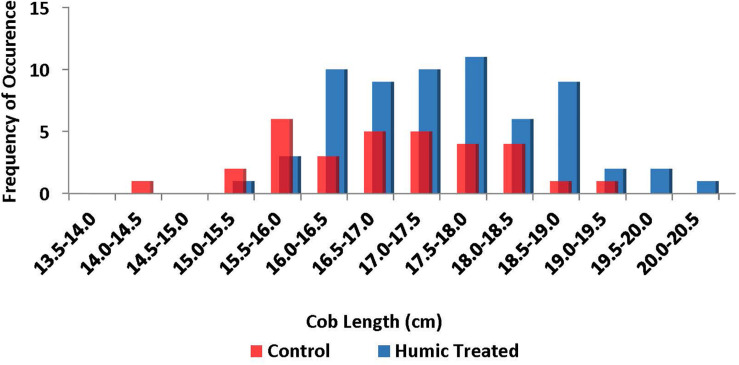
Distribution of cob lengths at physiological maturity for plant samples hand-collected from all treatment strips in all 4 years. Both application rates of the humic product are grouped together into “Humic Treated.” Total number of samples is 32 for the control and 64 for both humic product application rates combined.

Comparing their proportional increases with humic product application, grain weight increases in 2012 and 2016 appear to result primarily from increased cob length, while grain weight increases in 2013 and 2014 appear to reflect both cob length and 100-kernel weight. For all comparisons of humic product vs. control, the increase in mean cob length resulted mostly from a shift in proportions from the shorter side to the longer side of cob lengths (15.0 – 19.5 cm) that were achieved by both the control and humic treatments (Figure 4). Only a modest proportion of the increase in mean cob length appears to result from cob lengths increasing to high values (19.5–20.5 cm) not reached by any control samples.

### Grain Quality

Grain quality determination during the 2012–2014 seasons found that grain contents of protein, starch, and oil showed only few significant responses to humic product application. In the 2012 field season, the 3 L ha^–1^ split application of the humic product caused a significant (*P* = 0.06) decrease in grain protein content for the upland transect from 75.1 g kg^–1^ (control) to 70.2 g kg^–1^, while accompanying decreases in the lowland transect for both humic product treatments (from 74.5 g kg^–1^ to 71.5 g kg^–1^) were insignificant (*P* = 0.67). In the upland transect the 3 L ha^–1^ split application treatment also caused a significant (*P* = 0.097) increase in starch content from 612 g kg^–1^ to 617 g kg^–1^. Starch concentration increased non-significantly for this treatment in the lowland transect and for the single application in both transects (data not shown). In 2013, both humic product treatments caused significant decreases in protein content for the upland transect (both *P* < 0.05), although both also caused non-significant protein increases in the lowland transect. Numeric increases in starch content with humic product application in the upland transect were insignificant (data not shown), while starch content decreases in the lowland transect were significant (*P* = 0.096) for the 3 L ha^–1^ split application. No significant responses to the humic product occurred in 2014 for protein, starch or oil contents (data not shown). In summary, a slight shift from protein toward starch accumulation likely occurred in the upland transect during the droughtier two of the three seasons, and the shifts were more pronounced in the droughty 2012 season. Elsewhere, humic product application in field conditions was also associated with increased carbohydrate accumulation by potato (*Solanum tuberosum* L.) (Selim et al., 2009) and sweet potato (*Ipomoea batatas* L.) (El-Sayed et al., 2011).

### Plant Nutrients

Nutrient concentrations for young leaves sampled at the R2 reproductive growth stage were within acceptable ranges for normal maize growth (data not shown, Bryson and Mills, 2014) in at least two of the three 2012–2014 seasons for all nutrients except mild S and Zn deficiencies, which occurred in all three seasons and in both landscapes. Young leaf nutrients were not measured in 2016 due to the lack of consistent responses to humic treatments in the previous 3 years.

Across all 3 years, nutrient concentrations for all three leaf samplings and grain and stover at physiological maturity showed few significant responses to the humic product, none of which was broadly consistent across treatments, soil types/landscapes, and years. For example, simultaneous with the large grain yield responses to the humic product in 2012, the only significant response of grain nutrient concentrations to either humic application rate was decreased N in the upland transect (Supplementary Table 1). For stover nutrient concentrations, in the upland transect only B increased significantly, and in the lowland transect only P and K increased (Supplementary Table 2). Specific responses of young leaf nutrients for the 2012 season are discussed in the Supplementary Material. In summary, none of the trends found for one crop part in 2012 was reproduced for the other two crop parts.

This specific array of significant responses in 2012 was not reproduced in 2013, 2014, or 2016, which instead provided unrelated patterns of similarly scattered responses. Their details are discussed in the Supplementary Material.

In the two drier years of 2012 and 2013, B was a relatively responsive nutrient to both humic product treatments, mostly in the upland landscape. Further details are discussed in the Supplementary Material. We do not view enhanced B uptake as a mechanistic explanation for positive crop responses to humic products.

In summary, plant nutrient concentrations responded irregularly and inconsistently to the humic product across years, soil types/landscapes, and plant parts. This randomness suggests enhanced availability and uptake of soil nutrients was not the causal mechanism for crop responses to the humic product that were observed in this study. Similarly, we note that the greater incidence of negative nutrient responses to the humic product in 2014, as opposed to greater incidences of positive nutrient responses in the drier years, did not inhibit crop growth in 2014.

### Leaf Area

For the Ames field, total leaf area increased above the control with humic product application by small positive percentages in 10 of 12 cases for all soil type/landscape by treatment combinations in 2012, 2014, and 2016 (Table 6). The highest percent increase was 5.3% for the 3 L ha^–1^ split application treatment in the lowland transect of 2012. Across both landscapes, total leaf area did not respond significantly to either humic product application rate in any year, while soil type/landscape effect was significant only in 2014, with greater values in the lowland landscape. Within either soil type/landscape, total leaf area response to either application rate approached significance only in 2012 for both humic treatments in the lowland transect: *P* = 0.11 for the single application and *P* = 0.02 for the split application. Responses of individual leaf areas to the humic product are presented in the Supplementary Material.

**TABLE 6:** Total maize leaf area responses to humic product application for the Ames on-farm field trial (2012, 2014, and 2016) and for the Kelley on-farm field (2013).

2012 (Ames field)							

Total leaf area (cm^2^)							

Treatment	Upland^a^	Lowland^b^	Mean	Statistics:	LSD Pr > F^c^	Tests	LSD Pr > F
C	6827	6817	6821	Treatment	0.26	C vs. H1	0.51
H1^*d*^	6820	7043	6932	Landscape	0.18	C vs. H2	0.19
H2^*e*^	6912	7176	7044	Interaction	0.85	H1 vs. H2	0.48
Mean	6853	7012	6933				

	**By upland**				**By lowland**		
	**Tests**	**LSD Pr > F**			**Tests**	**LSD Pr > F**	

	C vs. H1	0.75			C vs. H1	0.11	
	C vs. H2	0.50			C vs. H2	0.02	
	H1 vs. H2	0.69			H1 vs. H2	0.33	

**2014 (Ames field)**							

**Treatment**	**Upland**	**Lowland**	**Mean**	**Statistics:**	**LSD Pr > F**	**Tests**	**LSD Pr > F**

C	6442	6846	6644	Treatment	0.21	C vs. H1	0.29
H1	6616	7045	6830	Landscape	<0.01	C vs. H2	0.26
H2	6593	7092	6842	Interaction	0.99	H1 vs. H2	0.94
Mean	6550	6994	6772				

	**By upland**				**By lowland**		
	**Tests**	**LSD Pr > F**			**Tests**	**LSD Pr > F**	

	C vs. H1	0.48			C vs. H1	0.44	
	C vs. H2	0.54			C vs. H2	0.34	
	H1 vs. H2	0.92			H1 vs. H2	0.85	

**2016 (Ames field)**							

**Treatment**	**Upland**	**Lowland**	**Mean**	**Statistics:**	**LSD Pr > F**	**Tests**	**LSD Pr > F**

C	6703	6938	6821	Treatment	0.86	C vs. H1	0.71
H1^*f*^	6827	6991	6909	Landscape	0.32	C vs. H2	0.94
H2^*g*^	6883	6720	6802	Interaction	0.59	H1 vs. H2	0.65
Mean	6804	6883	6844				

	**By upland**				**By lowland**		
	**Tests**	**LSD Pr > F**			**Tests**	**LSD Pr > F**	

	C vs. H1	0.61			C vs. H1	0.87	
	C vs. H2	0.47			C vs. H2	0.50	
	H1 vs. H2	0.82			H1 vs. H2	0.41	

**2013 (Kelley field)**							

**Treatment**	**Upland^*a*^**	**Lowland^*h*^**	**Mean**	**Statistics:**	**LSD Pr > F**	**Tests**	**LSD Pr > F**

C	6306	6574	6440	Treatment	0.22	C vs. H1	0.34
H1^*d*^	6497	6772	6635	Landscape	0.08	C vs. H2	0.09
H2^*e*^	6648	6958	6804	Interaction	0.99	H1 vs. H2	0.40
Mean	6484	6768	6626				

	**By upland**				**By lowland**	
	**Tests**	**LSD Pr > F**			**Tests**	**LSD Pr > F**

	C vs. H1	0.39			C vs. H1	0.45
	C vs. H2	0.14			C vs. H2	0.17
	H1 vs. H2	0.49			H1 vs. H2	0.48

*Two rates of humic product application (H1 and H2) were compared to an unamended control (C).*

*^a^Clarion loam.*

*^b^Webster silty clay loam.*

*^c^Probability of statistical significance as determined by the Least Significance Difference method.*

*^d^Enersol humic product broadcast applied in 2012 and 2014 at 2.5 L ha^–1^ at the maize fourth leaf stage.*

*^e^Enersol humic product broadcast split-applied in 2012 and 2014 at 2.0 L ha^–1^ at maize post-planting before pre-emergence and 1 L ha^–1^ at the maize fourth leaf stage.*

*^f^Enersol humic product broadcast applied in 2016 at 2.3 L ha^–1^ at the maize fourth leaf stage.*

*^g^Enersol humic product broadcast applied in 2016 at 4.7 L ha^–1^ at the maize fourth leaf stage.*

*^h^Canisteo silty clay loam/Harps loam.*

For the Kelley field (2013), across both humic treatments total area of the 19 leaves was significantly greater (*P* = 0.08) in the lowland transect than in the upland transect (Table 6). Increases in total leaf area above the control were modest positive percentages for all four combinations of soil type/landscape by humic treatment. The highest percent increases were 5.8% and 5.4% for the 3 L ha^–1^ split application treatment in the lowland and upland transects, respectively. Across both transects, total leaf area responded significantly (*P* = 0.09) to the 3 L ha^–1^ split application treatment. Responses of individual leaf areas to the humic product are presented in the Supplementary Material.

Summarizing all four seasons, the largest proportional increases in total leaf area with humic product application occurred in the droughtier 2012 and 2013 seasons. The corresponding levels of significance were generally numerically greater (smaller P levels) than those of 2014 and 2016 (Table 6).

### Soil Properties

Across all 4 years, the lowland transect had greater concentrations of soil nutrients in the vast majority of comparisons with the upland transect (Supplementary Tables 7–10). Individual comparisons varied substantially among the years, though, suggesting random variation across time in either soil sampling and/or laboratory analyses. Manure application to the Ames field prior to the 2016 season (Supplementary Table 10) resulted in moderate to large numeric increases above the 2014 levels (Supplementary Table 9) for nearly all extractable nutrients other than Mn. Corresponding increases for SOM, CEC, and both pH parameters were muted or absent.

When partitioned by soil type/landscape within each year, soil properties in either humic product treatment differed significantly (*P* < 0.10) from the control in only 14 of 240 cases for all four growing seasons (Supplementary Tables 7–10). Of these differences, 13 were increases above the control. The 14 cases involved 10 soil properties, indicating inconsistent soil responses across soil types/landscapes and years. In short, soil properties showed no meaningful responses to humic product application.

## Discussion

Crop responses to agricultural inputs often vary across soil types/landscapes and time, and much research has sought to identify systematic causes of those variations in order to develop wiser management of the inputs. For example, variability in plant uptake of N and other nutrients was one rationale for development of site-specific or precision farming technologies (Pierce and Nowak, 1999). That a variable response exists across soil types/landscapes or time clearly does not exclude an agricultural input from being a viable tool for crop production. Similarly, inconsistent crop responses to humic product applications, as suggested by previous studies, do not justify a conclusion that humic products are unreliable. Instead, understanding the process-level causes of their variable effects becomes a worthy research objective. A first step toward that objective is to identify spatial and temporal patterns in their field efficacy.

The results of this study suggest that specific factors can be identified to explain variable crop responses to humic products. In our study, maize growth and grain yield responses to a humic product varied across time and space gradients within a high-yielding region. During the severe drought of 2012, grain yield responses to the humic product in the Ames field differed systemically among soil types/landscape positions and their associated soil types: the greatest gains in maize grain yield with the humic product occurred where the effects of drought should be most pronounced – the eroded upland soil with the coarsest texture and presumably lowest soil water availability compared to the sideslope and lowland areas. There was still significant benefit to maize grain yield with the lower rate of humic product application at the lowland landscape position. While we did not measure soil water relations during this study, we visually noted in the 2012 growing season that (i) the lowland landscape position had wetter soil conditions than did the upslope positions, and (ii) its onset of crop drought symptoms was delayed compared to the upland.

Enhancement of crop response to the humic product under droughtier conditions was also evident across growing seasons. The most pronounced responses in combine grain yield, hand-sampled grain weight, cob length, and total leaf area were in the droughtier years of 2012 and 2013, especially in the upland transects. In 2014 and 2016, by contrast, abundant rainfall and hence little environmental stress in this high-yield setting led to subdued maize growth responses. Numerically positive responses of several growth parameters to the humic product were commonly observed in all years and soil types/landscape positions, but they were more likely to reach statistical significance in the droughtier years and the upland landscape.

Partial alleviation of drought stress through application of humic materials, as suggested in our study, has already been demonstrated in controlled conditions, for example by studying maize seedlings (Bijanzadeh et al., 2019; Canellas et al., 2020), maize growth (Anjum et al., 2011) and creeping bentgrass (*Agrostis stolonifera*, L.) (Zhang and Ervin, 2004). In field studies, crops being grown at suboptimal irrigation water rates responded significantly to humic product application for wheat (*Triticum* sp.) in Iran (Shahryari et al., 2012), barley (*Hordeum vulgare* L.) in Saudi Arabia (Almarshadi and Ismail, 2014), and pear (*Pyrus communis* L.) in Egypt (Ismail et al., 2007).

Our rainfed location did not allow such a design. Instead, we coarsely quantified the relationship of humic product use to drought stress alleviation by correlating (1) crop growth responses to humic product use in the upland transects in each of the 4 years to (2) the total precipitation amount during each growing season (April-September) expressed as a ratio to the 30-year (1981–2010) precipitation means for those months. Increasing drought stress would be represented as decreasing precipitation ratios. Including both humic product treatments in the correlation resulted in correlation coefficients of −0.609 (*P* = 0.109) for combine grain yield, −0.669 (*P* = 0.0697) for cob length, −0.390 (*P* = 0.339) for grain weight in the yield components, −0.338 (*P* = 0.413) for stover weight, and 0.403 (*P* = 0.323) for total leaf area (Supplementary Table 11). The negative correlation coefficients are consistent with drought stress (decreasing precipitation ratios) causing greater crop response to humic product use. The near significance of the combine grain yield correlation supports the association of humic product use with drought stress alleviation, as does the significance of the cob length correlation. The correlation of the yield component grain weight was improved to −0.947 (*P* = 0.0012) through deletion of the grain weight in the lowest yielding humic product treatment in the 4-year study, the 2.5 L ha^–1^ treatment in 2012. One possible explanation for this improved correlation is our observation that locating representative yield component samples was most difficult in low-yielding plots, as our yield component samplings were restricted to plants that were evenly spaced and had developed healthy, filled ears, even if this was not representative of growth throughout the low-yielding plots. Hence especially for low-yielding plots, we believe the combine grain yield trends are more reliable than are yield component trends. We did not attempt similar correlations for the lowland transects, where crop growth was less vulnerable to annual variations in drought stress.

We report these numeric responses by individual soil type/landscape even when the soil type/landscape effect was insignificant at *P* < 0.10. Selecting the threshold of significance in field work involves some discretion: milder thresholds than *P* < 0.10 are also justifiable (Carmer, 1976). At P levels less stringent than 0.10, more of our soil type/landscape effects and plant measurements would become significant. Hence their individual description can provide useful information, especially to researchers in other regions with less favorable growing conditions. For example, describing results of the upland landscapes each year emphasizes the large effect of annual precipitation, which will be useful for corn production in drought-prone regions. The consistency of small positive responses for most plant parameters is striking, and their magnitude differed numerically between soil types/landscapes and among years in a predictable manner. Reporting numerically consistent trends for individual year by soil type/landscape combinations enables the useful conclusion that this humic product has the capability to improve crop growth in field conditions, but significance at our *P*-value of 0.10 was reached only in droughtier conditions. Describing the incidences of these small responses by soil type/landscape is useful for understanding when and where a humic product is more likely to be effective, which is the main objective of site-specific management. It also guides our further research into mechanistic investigations, which we will report subsequently.

Annual changes in the maize cultivars cannot explain these variable crop responses to the humic product. The whole of each field was planted to only one cultivar in each year, yet in the drier 2012 and 2013 seasons the droughtier upland transect provided stronger crop responses than did the lowland transect.

That stress alleviation is a central reason for favorable crop responses to this humic product was further suggested by the finding that the grain yield response resulted primarily from a reduction in the number of shorter ears (Figure 4). In other words, the grain yield boost was achieved largely by enhancing growth of the weaker plants. This preferential support of smaller plants is also a form of environmental stress alleviation, in that the smaller plants would otherwise be disadvantaged in their competition against their larger neighbors for light, water, and nutrients, given the high population stands that characterize Corn Belt production.

This study was designed to establish the degree that environmental factors impact humic product efficacy in representative on-farm conditions. Its emphasis on field measurements was not suitable for identifying causal mechanism(s) of crop responses. Nevertheless, our results speak against the primary mechanism being nutrient-based. Positive responses of individual nutrient concentrations to humic product application were infrequent and inconsistent across nutrients, years, and soil types/landscapes, speaking against any single nutrient as the key mechanism. Young leaf nutrient concentrations at the R2 stage indicated that S and Zn were the only nutrient deficiencies that occurred in each of the 2012–2014 seasons. With humic product application, neither concentration of these limiting nutrients increased significantly in this young leaf sampling except for Zn in 2014, when the crop did not respond to humic product application. Sulfur and Zn concentrations in grain and stover increased sporadically and inconsistently with humic product application. Soil nutrients showed no consistent responses to humic product application. Manure application to the Ames field prior to the 2016 season caused large numeric increases for extractability of nearly all soil nutrients. Despite these more fertile soil conditions, crop yield component responses to the humic product were slightly clearer in 2016 than in 2014. Thus, we hypothesize that the fundamental mechanism for plant responses to a humic product is unrelated to soil nutrient availability. The unidentified actual mechanism might, however, stimulate plant nutrient uptake as a secondary benefit by increasing plant nutrient demand. These observations are consistent with the widely held view of humic products as biostimulants, which promote plant growth through stimulation of cellular-level plant processes, as discussed by Nardi et al. (2002), Mora et al. (2010), Zandonadi et al. (2013), Berbara and Garcia (2014), Calvo et al. (2014), and Olaetxea et al. (2018).

This study demonstrated some difficulties in field evaluations. First, production fields often have multiple soil types, each of which might support differing conclusions. For example, the droughtier upland soils provided more significant crop responses to the humic product than did downslope soils. Conversely, the most negative crop response was in an overly wet setting, the lowland soil of 2013. Future research will explore further such incidences of negative crop responses in seasonally wet soils that we have observed elsewhere. Second, high replicate variability is a challenge that must be considered when designing adequate field designs. For example, maize growth was limited in the eroded replicate 4 of the Ames upland transect compared to the other three upland replicates (data not shown). The resulting variability inhibited the establishment of statistical significance for some crop responses, despite appreciable numeric differences. For example, in the upland transect of 2016, combine grain yield showed increases of less than 1% with both humic product treatment, while grain weight of the yield component samples showed increases of 3.8% and 6.2%, which better aligned with field observations. Yield component samplings could more easily be fitted into areas of representative crop growth compared to combine grain yield.

A third and broader challenge posed by this research locale was the high-yielding nature of maize production in central Iowa. Field-average grain yields of 9.4 to 14.4 Mg ha^–1^ obtained here for each year (or 149 to 229 bushels acre^–1^) surpassed the corresponding national average yields (USDA-National Agricultural Statistics Service, 2018) by 18 to 31% (mean 23%) for these years. The control treatments alone surpassed the national averages by 9 to 29% (mean 17%). This study reported significant crop responses to humic product application in cases despite these favorable conditions and high yield levels. Our mixed results confirm previous studies (Calvo et al., 2014) by suggesting that environmental stress mitigation is a large component of crop responses to humic products. Hence, field studies of humic products in settings less favorable than central Iowa might lead to yet more pronounced and frequent crop responses.

Our results demonstrate the capacity of the Enersol humic product to improve crop growth in field conditions, even in the high-yielding setting of the western Corn Belt. In addition, we also tested the hypothesis that environmental constraints predictably altered humic product efficacy. Our research measured multiple parameters both at in-season and harvest times, and they were repeated across time and space. The resulting large number of comparisons between an unamended control and humic product treatments allowed the nuanced observation that maize frequently showed positive but subtle growth responses to the humic product and that their magnitudes increased in droughtier settings. Given the vast potential array of environmental conditions, crop types and varied crop management practices, more such detailed studies are needed to fully assess field efficacies of humic products. As demand for increased crop production occurs over time with increasing global population, combined with diminishing availability of arable land, more pressure is exerted on marginally productive lands. Our research points to humic products as being a helpful tool in managing profitable crop production on those marginal lands, particularly where water is limiting to support crop production. This study also illustrates the rigor to which humic products should be evaluated. An adequate number of field replications is absolutely necessary to enable precise statistical analyses, and in-season plant analyses are necessary to depict the development of a grain yield response. Such information would more efficiently guide future research into the processes underlying crop responses to humic products.

## Conclusion

Application of the Enersol humic product during four maize seasons in production fields of central Iowa led in cases to significant increases in maize grain yield, ear length, stover weight, and leaf areas. These beneficial crop responses were most evident in droughtier settings: the 2012 and 2013 growing seasons, and in the upland transect with its coarser textured soil. The yield increase resulted mostly from smaller proportions of short ears. In a high-yielding crop region having little environmental stress other than drought, our results support earlier research findings from controlled conditions and field studies that humic products can benefit crop growth through alleviation of environmental stresses. This relationship would help explain inconsistencies among results obtained by multiple studies. Our results suggest a systematic pattern to the field efficacy of humic products. Our results are also consistent with but do not prove the view of humic products as biostimulants that enhance crop physiological processes.

## Data Availability Statement

The datasets generated for this study will be uploaded to the In-house Database Management System of the USDA-ARS National Laboratory for Agriculture and the Environment, from where it will be made available for all reasonable requests.

## Author Contributions

DD and DO contributed to the design of the study. DD and DO managed the field experiments and together with CH and GR oversaw the sample analyses. DO and DD developed the interpretations. DO and DD drafted the manuscript. All authors reviewed and approved the manuscript.

## Conflict of Interest

JS and JD were employed by the company Minerals Technologies, Inc. The authors declare that this study received funding from both USDA-ARS and the American Colloid Company through USDA-ARS Trust Fund Cooperative Agreement 58-3625-2-573. American Colloid Company agreed to the publication of this manuscript but was not involved in the study design, collection, analysis, interpretation of data, the writing of this article, or the decision to submit it for publication.

## Footnotes

1 Reference to any specific commercial product is for the information of the public and does not constitute endorsement or recommendation by the United States government or other sponsors of this report.

2 https://www.usclimatedata.com/climate/ames/iowa/united-states/usia0026/2017/1
