# Supplementary material for: Maize Growth and Grain Yield Responses to a Micronized Humic Product Across Soil Types and Annual Weather Patterns in Central Iowa, United States
Source: Front Plant Sci. 2021 May 12;12:672078. doi: 10.3389/fpls.2021.672078 (PMC8153041; doi:10.3389/fpls.2021.672078)
Supplement: Supplementary file 1 [file Data_Sheet_1.docx]

**SUPPLEMENTARY MATERIAL**

**Plant Nutrient Concentrations**

Specific responses of young leaf nutrients to humic product application in 2012 are as follows. Leaf sampling at the 14^th^ -15^th^ leaf growth stage--the time of maximum daily nutrient uptake—gave scattered results. Potassium increased significantly in the upland transect with one humic product treatment (and it increased numerically at the other two leaf sampling times, data not shown), but it decreased nearly significantly (P=0.10) in the lowland transect (Table S3). Also in the lowland transect, Ca increased significantly for one humic treatment and increased Fe nearly reached significance, but Mn decreased nearly significantly. No other nutrient responses were significant for young leaf nutrients at the 14^th^ – 15^th^ leaf stage. For the 10^th^ leaf stage sampling, P increased significantly with one humic treatment in the upland transect but N, S, Cu, and Fe decreased significantly with one humic treatment in the lowland transect. No nutrients responded significantly at the R2 leaf sampling time.

For grain nutrients in 2013, in the upland transect P, Mg, Fe, and B had significant positive responses to mostly one humic product treatment, and in the lowland transect P, Mg, K, S Cu, and N responded positively to mostly one humic product treatment (data not shown). For stover nutrients, in the upland transect Mg, Ca, S, and Mn responded positively to mostly one humic product treatment (data not shown). For leaf nutrients, in the upland transect N increased at both the V10 and R2 samplings and Mn responded at the V14 sampling (data not shown).

In 2014, the only significant grain nutrient responses to either humic product treatment were negative for S in both landscapes, Cu in the lowland and B in the upland transect (Table S4). For stover nutrients, K, Mn, and Cu were significantly lower for one humic product treatment in the lowland transect and Zn had a negative response in the upland transect (Table S5). Only Fe increased significantly, in the upland transect. At P<0.20, additional negative responses to one humic product treatment in the lowland transect occurred for P, K, Ca and Fe, while in the upland transect B increased. For young leaf nutrients at the 14^th^ to 15^th^ leaf stage, P and B responded positively to one or two humic product treatments, respectively (Table S6). For the other two sampling times of young leaves, the only significant nutrient response to either humic product treatment was decreased Cu in the upland transect at the R2 sampling time.

In 2016, the only significant responses of grain nutrients were all for the 4.7 L ha^-1^ treatment, namely positive for Zn in the lowland transect and negative for Cu and B in the upland transect (data not shown). For stover nutrients, the sole significant responses to either humic product were that Zn increased in the lowland transect, while N decreased in the lowland and Cu decreased in the upland transects (data not shown).

Boron showed significant positive responses to either humic product treatment in the upland transect for 2012 stover (Table S2), 2013 grain and also young leaf samplings (data not shown), and in 2014 for the 14^th^ to 15^th^ leaf stage sampling (Table S6). Boron also increased numerically but nonsignificantly for either humic product treatment for 2012 grain, 2013 stover, 2014 stover, and 2016 stover in both transects and 2013 stover in the upland transect. Yet grain B decreased significantly in response to either humic product treatment in both 2014 in the upland transect and 2016 across both transects.

In the two drier years of 2012 and 2013, B concentrations were significantly (often highly significantly) less in the upland transect than in the lowland transect in 2012 for grain (Table S1), the 14^th^ to 15^th^ leaf stage sampling (Table S3) and the other two 2012 leaf samplings (data not shown), and in 2013 for grain, stover and all young leaf samplings (data not shown). Similar to B trends in 2012, the lowland transect had greater B concentrations than did the upland transect for 2014 stover (Table S5), the 14^th^ to 15^th^ leaf stage sampling (Table S6) and the other two 2014 leaf samplings (data not shown). Yet the upland transect had significantly greater B concentrations than did the lowland transect for 2016 grain and stover (data not shown), while no landscape trend was clear for 2014 grain.

The dominant B species in soil is the neutral B(OH)_3_, which might primarily enter the plant through passive flow (Brown et al., 2002). Its availability might therefore in concept be associated with crop uptake of soil water, as B deficiencies have been reported in droughty conditions (Marschner, 1986). This association would explain the lower B concentrations in the upland transect than in the lowland transect in the droughtier 2012 and 2013 growing seasons. We do not have a clear explanation for its frequent responses to humic product application, other than the speculation that increased plant growth with humic product application should also lead to greater uptake of soil water and hence B. We do not view enhanced B uptake as a potential mechanistic explanation for positive crop responses to humic products.

**Plant Leaf Area**

For the Ames field, landscape significantly affected individual leaf area for about one-third of all leaves in 2012, 2014, and 2016. Hence, separating the transects for each of the 19 leaves in 2012 and 2014 and each of the 20 leaves in 2016, individual leaf areas that increased significantly with either humic treatment, starting with the third leaf, were: (i) in 2012 leaves 3, 12, and 13 for the upland transect, leaves 7, 9, 10, and 11 for the lowland transect, and leaf 6 for both transects; (ii) in 2014 leaves 14 and 17 for the upland transect, leaves 4, 6, 8, and 9 for the lowland transect, and leaf 3 for both transects; and (iii) in 2016 leaves 5 and 6 for the lowland transect (data not shown).

Regarding individual leaf areas in the Kelley field (2013), the main humic product treatment across both transects was significant for leaves 12 to 14 (data not shown). Leaves that responded significantly for either humic treatment across both transects were leaves 6, 12, 13, and 14 for the 3.0 L ha^-1^ split application, and leaf 13 for the 2.5 L ha^-1^ single application (data not shown). Landscape significantly affected area for leaves 2 to 7, 13, and 14. Hence examining each transect separately, a positive leaf area response to the 3 L ha^-1^ split application treatment was significant for leaves 5 and 13 to 15 in the upland transect and for leaves 12 to 14 in the lowland transect (data not shown). Positive leaf area response to the 2.5 L ha^-1^ application treatment reached significance for leaves 11, 13 and 15 in the upland transect and for leaf 12 in the lowland transect. Also for the upland transect, leaf response approached significance (0.10<P<0.15) for leaves 2 to 4, 7, and 16 with the 3 L ha^-1^ split application treatment and for leaves 2 and 14 with the 2.5 L ha^-1^ application treatment (data not shown). No other leaf responses approached significance.

**REFERENCES**

Brown, P.H., Bellaloui, N., Wimmer, M.A., Bassil, E.S., Ruiz, J., Hu, H., Pfeffer, H., Dannel, F., and Römheld, V. (2002). Boron in plant biology. *Plant Biol*. 4, 205-223.

Marschner, H. (1986). *Mineral Nutrition in Higher Plants*. London: Academic Press.

Table S1. Total nutrient concentrations for maize grain at physiological maturity in the 2012 Ames field for the 2.5 L ha^-1^

single application (HP1) and 3.0 L ha^-1^ split application (HP2) of the Enersol humic product. Levels of significance (Pr)

are for each humic product treatment against the unamended control within each landscape.

| Landscape | Treatment | N | P | K | Mg | Ca | S | Zn | Mn | Cu | Fe | B |
| --- | --- | --- | --- | --- | --- | --- | --- | --- | --- | --- | --- | --- |
|  |  | -------------------------------g kg^-1^--------------------------------- | | | | | | -----------------------mg kg^-1^----------------------- | | | | |
|  |  |  |  |  |  |  |  |  |  |  |  |  |
| Upland | Control | 11.8 | 1.81 | 3.4 | 0.88 | 0.35 | 0.75 | 22.7 | 2.52 | 1.83 | 95.0 | 3.46 |
|  | HP1 | 11.8 | 2.05 | 3.6 | 0.93 | 0.30 | 0.75 | 27.4 | 3.81 | 1.80 | 128.1 | 3.70 |
|  | HP2 | 10.6 | 1.95 | 3.8 | 0.90 | 0.45 | 0.75 | 19.5 | 2.01 | 1.90 | 85.6 | 3.99 |
|  |  |  |  |  |  |  |  |  |  |  |  |  |
|  | Pr of HP1 | 0.80 | 0.37 | 0.27 | 0.27 | 0.14 | 0.37 | 0.30 | 0.80 | 0.83 | 0.43 | 0.37 |
|  | Pr of HP2 | <0.01 | 0.65 | 0.82 | 0.59 | 0.16 | 0.90 | 0.60 | 0.50 | 0.59 | 0.98 | 0.17 |
|  |  |  |  |  |  |  |  |  |  |  |  |  |
| Lowland | Control | 11.4 | 2.09 | 3.6 | 0.93 | 0.35 | 0.75 | 30.7 | 1.90 | 1.88 | 57.1 | 4.30 |
|  | HP1 | 10.3 | 2.26 | 3.7 | 0.93 | 0.40 | 0.70 | 17.8 | 3.93 | 2.05 | 75.3 | 4.37 |
|  | HP2 | 10.8 | 2.20 | 3.7 | 0.90 | 0.33 | 0.70 | 26.0 | 4.38 | 2.01 | 23.8 | 5.14 |
|  |  |  |  |  |  |  |  |  |  |  |  |  |
|  | Pr of HP1 | 0.42 | 0.12 | 0.33 | 0.96 | 0.33 | 0.15 | 0.84 | 0.61 | 0.30 | 0.85 | 0.77 |
|  | Pr of HP2 | 0.37 | 0.29 | 0.44 | 0.57 | 0.99 | 0.20 | 0.56 | 0.17 | 0.44 | 0.11 | 0.40 |

Table S2. Total nutrient concentrations for maize stover at physiological maturity in the 2012 Ames field for the

2.5 L ha^-1^ single application (HP1) and 3.0 L ha^-1^ split application (HP2) of the Enersol humic product. Levels of

significance (Pr) are for each humic product treatment against the unamended control within each landscape.

| Landscape | Treatment | N | P | K | Mg | Ca | S | Zn | Mn | Cu | Fe | B |
| --- | --- | --- | --- | --- | --- | --- | --- | --- | --- | --- | --- | --- |
|  |  | ----------------------------------g kg^-1^------------------------------- | | | | | | -----------------------mg kg^-1^---------------------- | | | | |
| Upland | Control | 5.0 | 0.34 | 8.2 | 3.1 | 4.2 | 0.40 | 17.7 | 42.8 | 4.25 | 87.2 | 7.06 |
|  | HP1 | 5.1 | 0.46 | 8.8 | 3.0 | 3.9 | 0.38 | 18.4 | 31.5 | 4.06 | 72.2 | 7.34 |
|  | HP2 | 4.9 | 0.42 | 8.6 | 3.0 | 4.3 | 0.45 | 23.1 | 33.7 | 4.24 | 170.7 | 8.20 |
|  |  |  |  |  |  |  |  |  |  |  |  |  |
|  | Pr of HP1 | 0.86 | 0.12 | 0.38 | 0.50 | 0.11 | 0.56 | 0.65 | 0.52 | 0.67 | 0.69 | 0.65 |
|  | Pr of HP2 | 0.41 | 0.32 | 0.55 | 0.61 | 0.40 | 0.18 | 0.94 | 0.53 | 0.98 | 0.89 | 0.09 |
|  |  |  |  |  |  |  |  |  |  |  |  |  |
| Lowland | Control | 4.7 | 0.38 | 6.4 | 3.0 | 4.2 | 0.38 | 17.6 | 20.2 | 3.96 | 72.9 | 6.78 |
|  | HP1 | 4.3 | 0.46 | 7.1 | 3.1 | 3.8 | 0.35 | 14.5 | 13.0 | 3.53 | 65.6 | 7.52 |
|  | HP2 | 4.5 | 0.40 | 7.0 | 3.0 | 3.9 | 0.40 | 14.6 | 13.4 | 3.44 | 69.0 | 7.55 |
|  |  |  |  |  |  |  |  |  |  |  |  |  |
|  | Pr of HP1 | 0.14 | 0.02 | 0.13 | 0.86 | 0.18 | 0.51 | 0.16 | 0.02 | 0.37 | 0.38 | 0.35 |
|  | Pr of HP2 | 0.31 | 0.53 | 0.17 | 0.64 | 0.29 | 0.43 | 0.25 | 0.02 | 0.29 | 0.64 | 0.33 |

Table S3. Total nutrient concentrations for a young maize leaf at the 14^th^ to 15^th^ leaf stage in the 2012 Ames field for the

2.5 L ha^-1^ single application (HP1) and 3.0 L ha^-1^ split application (HP2) of a humic product. Levels of significance are

for each humic product treatment against the control, across both landscapes.

| Landscape | Treatment | N | P | K | Mg | Ca | S | Zn | Mn | Cu | Fe | B |
| --- | --- | --- | --- | --- | --- | --- | --- | --- | --- | --- | --- | --- |
|  |  | ---------------------------------g kg^-1^-------------------------------- | | | | | | -------------------------mg kg^-1^------------------------ | | | | |
| Upland | Control | 33.7 | 2.96 | 20.5 | 2.75 | 3.98 | 2.03 | 23.5 | 36.8 | 11.6 | 116 | 16.2 |
|  | HP1 | 34.1 | 3.10 | 22.5 | 2.58 | 3.78 | 2.00 | 25.1 | 41.5 | 11.4 | 108 | 16.6 |
|  | HP2 | 34.8 | 3.16 | 21.8 | 2.63 | 4.25 | 2.10 | 24.7 | 43.2 | 11.2 | 109 | 17.3 |
|  |  |  |  |  |  |  |  |  |  |  |  |  |
|  | Pr of HP1 | 0.84 | 0.53 | 0.06 | 0.32 | 0.37 | 0.81 | 0.37 | 0.46 | 0.77 | 0.50 | 0.92 |
|  | Pr of HP2 | 0.48 | 0.36 | 0.22 | 0.44 | 0.25 | 0.55 | 0.48 | 0.30 | 0.59 | 0.43 | 0.54 |
|  |  |  |  |  |  |  |  |  |  |  |  |  |
| Lowland | Control | 34.6 | 3.74 | 23.8 | 2.40 | 3.63 | 2.18 | 25.1 | 25.9 | 10.1 | 97 | 20.3 |
|  | HP1 | 36.6 | 3.67 | 22.0 | 2.35 | 3.98 | 2.08 | 24.6 | 19.5 | 10.6 | 115 | 21.5 |
|  | HP2 | 35.2 | 3.71 | 23.6 | 2.58 | 4.23 | 2.08 | 26.1 | 29.1 | 10.9 | 103 | 22.8 |
|  |  |  |  |  |  |  |  |  |  |  |  |  |
|  | Pr of HP1 | 0.17 | 0.74 | 0.10 | 0.69 | 0.18 | 0.35 | 0.73 | 0.10 | 0.52 | 0.11 | 0.66 |
|  | Pr of HP2 | 0.72 | 0.87 | 0.85 | 0.28 | 0.02 | 0.37 | 0.59 | 0.40 | 0.31 | 0.58 | 0.35 |

Table S4. Total nutrient concentrations for maize grain in the 2014 Ames field for the 2.5 L ha^-1^ single application

(HP1) and 3.0 L ha^-1^ split application (HP2) of the Enersol humic product. Levels of significance (Pr) are for each

humic product treatment against the unamended control within each landscape.

| Landscape | Treatment | N | P | K | Mg | Ca | S | Zn | Mn | Cu | Fe | B |
| --- | --- | --- | --- | --- | --- | --- | --- | --- | --- | --- | --- | --- |
|  |  | -------------------------------g kg^-1^--------------------------------- | | | | | | -----------------------mg kg^-1^----------------------- | | | | |
|  |  |  |  |  |  |  |  |  |  |  |  |  |
| Upland | Control | 11.3 | 2.01 | 3.60 | 0.78 | 0.10 | 0.55 | 11.1 | 3.64 | 2.26 | 18.0 | 1.38 |
|  | HP1 | 11.9 | 1.84 | 3.40 | 0.78 | 0.10 | 0.48 | 10.2 | 3.38 | 2.15 | 17.1 | 0.81 |
|  | HP2 | 11.2 | 1.97 | 3.43 | 0.80 | 0.10 | 0.50 | 10.6 | 3.23 | 2.28 | 16.1 | 1.16 |
|  |  |  |  |  |  |  |  |  |  |  |  |  |
|  | Pr of HP1 | 0.18 | 0.26 | 0.19 | 0.95 | 1.00 | 0.02 | 0.33 | 0.52 | 0.64 | 0.74 | 0.09 |
|  | Pr of HP2 | 0.94 | 0.79 | 0.41 | 0.76 | 1.00 | 0.12 | 0.59 | 0.35 | 0.95 | 0.32 | 0.63 |
|  |  |  |  |  |  |  |  |  |  |  |  |  |
| Lowland | Control | 11.1 | 2.34 | 3.87 | 0.82 | 0.10 | 0.53 | 11.8 | 3.35 | 2.47 | 15.0 | 0.96 |
|  | HP1 | 10.7 | 2.41 | 3.80 | 0.88 | 0.10 | 0.48 | 13.5 | 3.14 | 2.25 | 15.2 | 1.03 |
|  | HP2 | 11.5 | 2.30 | 3.80 | 0.88 | 0.12 | 0.55 | 10.5 | 3.21 | 1.98 | 17.1 | 0.76 |
|  |  |  |  |  |  |  |  |  |  |  |  |  |
|  | Pr of HP1 | 0.26 | 0.59 | 0.97 | 0.46 | 0.88 | 0.09 | 0.25 | 0.37 | 0.38 | 0.71 | 0.81 |
|  | Pr of HP2 | 0.22 | 0.82 | 0.97 | 0.40 | 0.26 | 0.49 | 0.39 | 0.54 | 0.09 | 0.11 | 0.49 |

Table S5. Total nutrient concentrations for maize stover at physiological maturity in the 2014 Ames field for the

2.5 L ha^-1^ single application (HP1) and 3.0 L ha^-1^ split application (HP2) of the Enersol humic product. Levels of

significance (Pr) are for each humic product treatment against the unamended control within each landscape.

| Landscape | Treatment | N | P | K | Mg | Ca | S | Zn | Mn | Cu | Fe | B |
| --- | --- | --- | --- | --- | --- | --- | --- | --- | --- | --- | --- | --- |
|  |  | ----------------------------------g kg^-1^------------------------------- | | | | | | -----------------------mg kg^-1^---------------------- | | | | |
| Upland | Control | 4.65 | 0.155 | 9.45 | 1.45 | 2.10 | 0.200 | 8.28 | 32.1 | 6.88 | 113 | 2.60 |
|  | HP1 | 4.62 | 0.135 | 9.48 | 1.52 | 2.10 | 0.225 | 5.04 | 35.4 | 7.10 | 143 | 3.20 |
|  | HP2 | 4.69 | 0.165 | 9.92 | 1.32 | 2.20 | 0.225 | 4.80 | 33.3 | 6.86 | 140 | 3.00 |
|  |  |  |  |  |  |  |  |  |  |  |  |  |
|  | Pr of HP1 | 0.93 | 0.60 | 0.97 | 0.68 | 1.00 | 0.57 | 0.09 | 0.35 | 0.69 | 0.06 | 0.14 |
|  | Pr of HP2 | 0.90 | 0.79 | 0.50 | 0.49 | 0.67 | 0.57 | 0.07 | 0.73 | 0.97 | 0.08 | 0.31 |
|  |  |  |  |  |  |  |  |  |  |  |  |  |
| Lowland | Control | 4.69 | 0.272 | 9.85 | 1.28 | 2.50 | 0.225 | 7.01 | 23.6 | 6.42 | 152 | 3.59 |
|  | HP1 | 4.69 | 0.187 | 9.22 | 1.20 | 2.05 | 0.200 | 5.62 | 20.1 | 5.46 | 125 | 4.32 |
|  | HP2 | 4.65 | 0.190 | 9.00 | 1.40 | 2.52 | 0.225 | 6.52 | 24.0 | 7.09 | 136 | 4.00 |
|  |  |  |  |  |  |  |  |  |  |  |  |  |
|  | Pr of HP1 | 0.99 | 0.31 | 0.12 | 0.59 | 0.14 | 0.54 | 0.42 | 0.07 | 0.09 | 0.12 | 0.39 |
|  | Pr of HP2 | 0.93 | 0.19 | 0.04 | 0.38 | 0.93 | 1.00 | 0.77 | 0.85 | 0.22 | 0.32 | 0.63 |

Table S6. Total nutrient concentrations for a young maize leaf at the 14^th^ to 15^th^ leaf stage in the 2014 Ames field for the

2.5 L ha^-1^ single application (HP1) and 3.0 L ha^-1^ split application (HP2) of a humic product. Levels of significance are

for each humic product treatment against the control, across both landscapes.

| Landscape | Treatment | N | P | K | Mg | Ca | S | Zn | Mn | Cu | Fe | B |
| --- | --- | --- | --- | --- | --- | --- | --- | --- | --- | --- | --- | --- |
|  |  | ---------------------------------g kg^-1^-------------------------------- | | | | | | -------------------------mg kg^-1^------------------------ | | | | |
| Upland | Control | 26.8 | 3.00 | 18.9 | 1.70 | 2.05 | 1.48 | 20.1 | 50.8 | 8.18 | 59.8 | 7.56 |
|  | HP1 | 27.0 | 3.13 | 20.4 | 1.62 | 2.15 | 1.55 | 22.4 | 53.7 | 8.43 | 63.6 | 7.93 |
|  | HP2 | 27.7 | 3.30 | 20.0 | 1.50 | 1.95 | 1.50 | 22.8 | 47.6 | 7.53 | 52.2 | 8.11 |
|  |  |  |  |  |  |  |  |  |  |  |  |  |
|  | Pr of HP1 | 0.88 | 0.09 | 0.18 | 0.91 | 0.75 | 0.58 | 0.08 | 0.99 | 0.98 | 0.73 | <0.01 |
|  | Pr of HP2 | 0.54 | 0.67 | 0.33 | 0.63 | 0.45 | 0.78 | 0.95 | 0.78 | 0.16 | 0.82 | <0.01 |
|  |  |  |  |  |  |  |  |  |  |  |  |  |
| Lowland | Control | 25.8 | 3.08 | 19.1 | 1.55 | 1.88 | 1.38 | 24.3 | 50.9 | 7.79 | 67.1 | 8.29 |
|  | HP1 | 26.1 | 3.15 | 18.6 | 1.55 | 1.88 | 1.40 | 22.8 | 41.4 | 7.10 | 58.5 | 8.19 |
|  | HP2 | 25.4 | 3.24 | 19.6 | 1.40 | 1.88 | 1.42 | 25.1 | 42.8 | 7.70 | 68.3 | 8.90 |
|  |  |  |  |  |  |  |  |  |  |  |  |  |
|  | Pr of HP1 | 0.83 | 0.73 | 0.98 | 0.91 | 0.75 | 0.60 | 0.97 | 0.65 | 0.31 | 0.92 | 0.28 |
|  | Pr of HP2 | 0.80 | 0.17 | 0.96 | 0.24 | 0.53 | 0.78 | 0.29 | 0.93 | 0.33 | 0.58 | 0.78 |

Table S7. Soil properties following two rates of humic product application (2.5 L ha^-1^ at the V4 growth stage, and 2.0 L ha^-1^ at crop pre-emergence + 1.0 L ha^-1^ at V4, or “3.0 L ha^-1^ split”) compared to the unamended control in the 2012 Ames field. Lower case “b” identifies those values for the humic treatments that differ significantly (P<0.10) from the corresponding control within the respective transects.

|  | Upland transect | | |  | Lowland transect | | |
| --- | --- | --- | --- | --- | --- | --- | --- |
|  | Control | 2.5 L ha^-1^ at V4 | 3.0 L ha^-1^ split |  | Control | 2.5 L ha^-1^ at V4 | 3.0 L ha^-1^ split |
| SOM ^a^ (g kg^-1^) | 26.0 | 31.0b | 26.8 |  | 24.8 | 24.2 | 24.8 |
| CEC ^a^ (cmol_c_ kg^-1^) | 13.4 | 13.4 | 12.7 |  | 13.3 | 13.3 | 13.6 |
| pH | 5.72 | 5.90 | 6.12 |  | 6.62 | 6.90 | 6.58 |
| Buffer pH | 6.95 | 7.05 | 7.15 |  | 7.38 | 7.42 | 7.32 |
|  | Extractable nutrients (mg kg^-1^) | | | | | | |
| Nitrate-N | 4.33 | 6.75 | 7.50 |  | 4.75 | 7.50b | 5.25 |
| P | 8.8 | 10.2 | 10.8 |  | 7.8 | 7.5 | 7.0 |
| K | 104 | 125b | 127b |  | 96 | 103 | 97 |
| Ca | 1602 | 1728 | 1680 |  | 1562 | 1998b | 1977 |
| Mg | 287 | 303 | 288 |  | 259 | 326 | 328b |
| S | 3.25 | 2.50 | 3.00 |  | 2.75 | 2.50 | 2.75 |
| Fe | 44.7 | 52.6 | 42.0 |  | 33.6 | 30.9 | 33.7 |
| Zn | 0.62 | 0.82b | 0.60 |  | 0.52 | 0.65 | 0.60 |
| Mn | 18.8 | 20.4 | 17.1 |  | 12.9 | 7.8 b | 11.4 |
| Cu | 1.35 | 1.58 | 1.42 |  | 1.32 | 1.35 | 1.38 |
| B | 0.250 | 0.250 | 0.250 |  | 0.250 | 0.325b | 0.300b |

^a^ Acronyms: SOM, soil organic matter; CEC, cation exchange capacity.

Table S8. Soil properties following two rates of humic product application (2.5 L ha^-1^ at the V4 growth stage, and 2.0 L ha^-1^ at crop pre-emergence + 1.0 L ha^-1^ at V4, or “3.0 L ha^-1^ split”) compared to the unamended control in the 2013 Kelley field. Lower case “b” identifies the sole value for the humic treatments that differs significantly (P<0.10) from the corresponding control within its transect.

|  | Upland transect | | |  | Lowland transect | | |
| --- | --- | --- | --- | --- | --- | --- | --- |
|  | Control | 2.5 L ha^-1^ at V4 | 3.0 L ha^-1^ split |  | Control | 2.5 L ha^-1^ at V4 | 3.0 L ha^-1^ split |
| SOM ^a^ (g kg^-1^) | 17.8 | 20.2 | 18.2 |  | 40.8 | 41.2 | 41.2 |
| CEC ^a^ (cmol_c_ kg^-1^) | 13.4 | 12.0 | 12.0 |  | 22.7 | 23.8 | 24.2 |
| pH | 6.52 | 6.25 | 6.40 |  | 7.35 | 7.38 | 7.32 |
| Buffer pH | 7.18 | 7.18 | 7.15 |  | 7.50 | 7.50 | 7.40 |
|  | Extractable nutrients (mg kg^-1^) | | | | | | |
| Nitrate-N | 12.8 | 13.2 | 9.3 |  | 12.0 | 17.2b | 14.0 |
| P | 16.0 | 12.8 | 24.5 |  | 29.0 | 24.5 | 23.0 |
| K | 138 | 130 | 143 |  | 211 | 201 | 219 |
| Ca | 2011 | 1716 | 1653 |  | 3838 | 4030 | 4004 |
| Mg | 174 | 210 | 209 |  | 349 | 364 | 386 |
| S | 4.75 | 4.50 | 3.75 |  | 4.33 | 4.25 | 4.50 |
| Fe | 27.4 | 27.0 | 23.2 |  | 31.6 | 31.0 | 34.3 |
| Zn | 0.28 | 0.22 | 0.30 |  | 0.80 | 0.80 | 0.85 |
| Mn | 9.3 | 8.0 | 10.3 |  | 5.3 | 6.3 | 6.4 |
| Cu | 0.82 | 0.82 | 0.90 |  | 1.82 | 1.72 | 2.00 |
| B | 0.225 | 0.225 | 0.225 |  | 0.600 | 0.525 | 0.550 |

^a^ Acronyms: SOM, soil organic matter; CEC, cation exchange capacity.

Table S9. Soil properties following two rates of humic product application (2.5 L ha^-1^ at the V4 growth stage, and 2.0 L ha^-1^ at crop pre-emergence + 1.0 L ha^-1^ at V4, or “3.0 L ha^-1^ split”) compared to the unamended control in the 2014 Ames field. Lower case “b” identifies those values for the humic treatments that differ significantly (P<0.10) from the corresponding control within the respective transects.

|  | Upland transect | | |  | Lowland transect | | |
| --- | --- | --- | --- | --- | --- | --- | --- |
|  | Control | 2.5 L ha^-1^ at V4 | 3.0 L ha^-1^ split |  | Control | 2.5 L ha^-1^ at V4 | 3.0 L ha^-1^ split |
| SOM ^a^ (g kg^-1^) | 26.0 | 25.0 | 26.5 |  | 33.0 | 30.8 | 31.0 |
| CEC ^a^ (cmol_c_ kg^-1^) | 12.5 | 10.9 | 12.6 |  | 16.3 | 14.7 | 16.2 |
| pH | 5.88 | 6.05 | 6.38b |  | 6.62 | 6.82 | 6.65 |
| Buffer pH | 6.92 | 7.02 | 7.23 |  | 7.35 | 7.45b | 7.35 |
|  | Extractable nutrients (mg kg^-1^) | | | | | | |
| Nitrate-N | 9.75 | 9.33 | 8.25 |  | 6.00 | 5.67 | 8.25 |
| P | 10.5 | 8.33 | 10.8 |  | 15.5 | 16.3 | 16.2 |
| K | 116 | 109 | 137 |  | 120 | 109 | 112 |
| Ca | 1478 | 1306 | 1551 |  | 2426 | 2251 | 2445 |
| Mg | 248 | 229 | 252 |  | 383 | 352 | 370 |
| S | 6.50 | 6.75 | 6.67 |  | 6.00 | 5.50 | 6.25 |
| Fe | 32.8 | 24.7 | 32.1 |  | 29.7 | 18.6 | 28.2 |
| Zn | 0.65 | 0.50 | 0.68 |  | 0.78 | 0.68 | 0.78 |
| Mn | 44.3 | 21.4 | 30.2 |  | 25.3 | 17.2 | 23.4 |
| Cu | 1.18 | 1.05 | 1.00 |  | 1.08 | 1.08 | 1.20 |
| B | 0.375 | 0.275 | 0.325 |  | 0.375 | 0.300 | 0.333 |

^a^ Acronyms: SOM, soil organic matter; CEC, cation exchange capacity.

Table S10. Soil properties following two rates of humic product application (2.3 L ha^-1^ or 4.7 L ha^-1^ at the V4 growth stage) compared to the unamended control in the 2016 Ames field. Lower case “b” identifies the sole value for the humic treatments that differs significantly (P<0.10) from the corresponding control within its transect.

|  | Upland transect | | |  | Lowland transect | | |
| --- | --- | --- | --- | --- | --- | --- | --- |
|  | Control | 2.3 L ha^-1^ at V4 | 4.7 L ha^-1^ at V4 |  | Control | 2.3 L ha^-1^ at V4 | 4.7 L ha^-1^ at V4 |
| SOM ^a^ (g kg^-1^) | 26.8 | 27.2 | 24.8 |  | 32.8 | 33.5 | 33.8 |
| CEC ^a^ (cmol_c_ kg^-1^) | 13.0 | 12.9 | 11.9 |  | 17.1 | 16.3 | 18.0 |
| pH | 6.02 | 6.12 | 6.38b |  | 6.60 | 6.72 | 6.70 |
| Buffer pH | 6.92 | 6.98 | 7.12 |  | 7.12 | 7.32 | 7.12 |
|  | Extractable nutrients (mg kg^-1^) | | | | | | |
| Nitrate-N | 18.2 | 15.8 | 16.2 |  | 13.2 | 14.0 | 16.5 |
| P | 17.2 | 18.0 | 15.2 |  | 37.5 | 36.8 | 37.8 |
| K | 128 | 144 | 131 |  | 132 | 145 | 144 |
| Ca | 1547 | 1580 | 1536 |  | 2380 | 2394 | 2549 |
| Mg | 257 | 270 | 254 |  | 371 | 380 | 388 |
| S | 6.50 | 7.75 | 6.75 |  | 8.25 | 8.25 | 9.00 |
| Fe | 35.5 | 39.2 | 28.6 |  | 43.5 | 34.3 | 51.9 |
| Zn | 0.72 | 0.80 | 0.58 |  | 1.10 | 0.98 | 1.05 |
| Mn | 20.7 | 23.2 | 16.4 |  | 12.0 | 10.4 | 12.4 |
| Cu | 1.00 | 1.62 | 1.08 |  | 1.33 | 1.60 | 1.30 |
| B | 0.700 | 0.800 | 0.575 |  | 0.250 | 0.250 | 0.325 |

^a^ Acronyms: SOM, soil organic matter; CEC, cation exchange capacity

Table S11. Pearson correlation coefficients and their levels of significance (P, Least Significant Difference) between (a) five plant growth responses above the Control treatment for two application rates of the humic product in the upland transects in each of the four growing seasons, and (b) drought stress, approximated by the ratio of total rainfall from April to September in each year to the 30-year mean (1981-2010) for total rainfall in the same months.

|  | Plant response | |  | Precipitation (mm) | | Ratio | Correlation coefficient | P |
| --- | --- | --- | --- | --- | --- | --- | --- | --- |
|  | Lower rate^a^ | Higher rate^a^ |  | Within season | 30-year mean |  |  |  |
| Combine grain yield (Mg ha^-1^) | | | | | | | | |
| 2012 | 0.93 | 1.60 |  | 314 | 670 | 0.47 | -0.6092 | 0.1089 |
| 2013 | 0.16 | 0.70 |  | 566 | 670 | 0.84 |  |  |
| 2014 | 0.57 | 1.02 |  | 812 | 670 | 1.21 |  |  |
| 2016 | 0.12 | 0.06 |  | 795 | 670 | 1.19 |  |  |
|  |  |  |  |  |  |  |  |  |
| Cob length (cm) | | | | | | | | |
| 2012 | 0.77 | 1.10 |  | 314 | 670 | 0.47 | -0.6689 | 0.0697 |
| 2013 | 0.62 | 0.49 |  | 566 | 670 | 0.84 |  |  |
| 2014 | 0.45 | 0.39 |  | 812 | 670 | 1.21 |  |  |
| 2016 | 0.60 | 0.80 |  | 795 | 670 | 1.19 |  |  |
|  |  |  |  |  |  |  |  |  |
| Grain weight (Mg ha^-1^) | | | | | | | | |
| 2012 | 0.28 | 1.88 |  | 314 | 670 | 0.47 | -0.3881 | 0.3420 |
| 2013 | 1.20 | 1.13 |  | 566 | 670 | 0.84 |  |  |
| 2014 | 0.52 | 0.59 |  | 812 | 670 | 1.21 |  |  |
| 2016 | 0.63 | 1.01 |  | 795 | 670 | 1.19 |  |  |
|  |  |  |  |  |  |  |  |  |
| Stover weight (Mg ha^-1^) | | | | | | | | |
| 2012 | 0.61 | 1.07 |  | 314 | 670 | 0.47 | -0.3376 | 0.4134 |
| 2013 | 0.34 | 0.07 |  | 566 | 670 | 0.84 |  |  |
| 2014 | 0.34 | -0.14 |  | 812 | 670 | 1.21 |  |  |
| 2016 | 0.72 | 0.94 |  | 795 | 670 | 1.19 |  |  |
|  |  |  |  |  |  |  |  |  |
| Total leaf area (cm^2^) | | | | | | | | |
| 2012 | -7 | 85 |  | 314 | 670 | 0.47 | +0.4027 | 0.3227 |
| 2013 | 191 | 342 |  | 566 | 670 | 0.84 |  |  |
| 2014 | 174 | 151 |  | 812 | 670 | 1.21 |  |  |
| 2016 | 124 | 180 |  | 795 | 670 | 1.19 |  |  |

^a^ Lower application rate of humic product was a single application of 2.5 L ha^-1^ in 2012 to 2014 and of 2.3 L ha^-1^ in 2016. Higher application rate was a split application totaling 3.0 L ha^-1^ in 2012 to 2014 and a single application of 4.7 L ha^-1^ in 2016.
